# Supporting recovery: Intersections of health, belonging, and help seeking after a campus mass casualty event

**DOI:** 10.1371/journal.pone.0353453

**Published:** 2026-07-29

**Authors:** Sharon Jalene, Jason A. Ciccotelli, Lauren Gatto, Christian Ison, Jaime Carbajal, Andrew Hooyman

**Affiliations:** 1 Applied Health Sciences, School of Integrated Health Sciences, University of Nevada, Las Vegas, Las Vegas, Nevada, United States of America; 2 Department of Physical Therapy, School of Integrated Health Sciences, University of Nevada, Las Vegas, Las Vegas, Nevada, United States of America; 3 Department of Student Success, Office of the Associate Vice Provost for Student and Academic Success, University of Nevada, Las Vegas, Las Vegas, Nevada, United States of America; 4 Physical Therapy, Crean College of Health and Behavioral Sciences, Chapman University, Orange, California, United States of America; Nanjing University, CHINA

## Abstract

Mass casualty events (MCE), such as campus shootings, can severely impact the mental health of students, faculty, and staff. This study examined the psychological effects of an MCE at a minority-serving institution and explored how sense of belonging, cardiorespiratory fitness, and treatment preferences influenced recovery. To measure the impact of the MCE on mental health an anonymous survey was administered four to five months post-MCE to university students, faculty and staff. Within the survey, participants were asked to retrospectively report their depression (PHQ-9) and anxiety (GAD-7) symptoms as they recalled them two weeks prior to the MCE**,** and as they experienced them in the two weeks preceding survey completion. The survey also included current measures of sense of belonging (SBS), perceived discrimination (PEDQ), estimated cardiorespiratory fitness (eCRF), and intervention preference to cope with the impacts of the MCE. Participants reported significant perceived increases in PHQ-9 and GAD-7 scores from the pre-MCE recall to current time-periods. Increases in depression (PHQ-9 scores) from pre-MCE to recent recall were larger among respondents with lower pre-MCE anxiety (GAD-7 scores), higher perceived discrimination (PEDQ), and those currently receiving treatment for anxiety or depression; respondents not receiving treatment showed no significant change. Among students, lower belonging scores were associated with greater increases in depression. Neither belonging nor perceived discrimination were strongly linked to individual variables, indicating their multifactorial nature. Most participants reported a preference to learning about alternative interventions such as exercise or meditation over pursuing university counseling services.

## Introduction

From the 1960s to the end of 2023, thirteen college campuses in the United States were targets of mass shootings resulting in 102 deaths, as well as numerous physical, and emotional injuries [1]. For the purposes of this study, a mass shooting is referred to as a mass casualty event (MCE) resulting in three or more deaths, not including that of the shooter [2,3]. Additionally, reports indicate that as of December 17, 2024, since the start of the new year, there were 83 school shootings reported in the United States of which 27 occurred on college or university campuses [4]. These were not considered mass shootings due to the number of fatalities. All of these incidents shock public consciousness and traumatize students, employees, and communities. Long after the news cycle has passed, structural damage must be repaired while the college administration attempts to mitigate responses to the trauma such as anxiety and depression [5,6]. Regardless of location and cohort characteristics, suffering and grief affect all communities.

Without consideration of these incidents, college students report increased levels of depression and anxiety compared to non-academic peers [7,8], including reports that 38% of students suffer from depression and 34% anxiety [9] compared to 8.3% and 30% of adult Americans, respectively [10]. This is attributed to a variety of factors, such as increased stress, lack of sleep, history of perceived discrimination, separation from home, and adjustments to independent living [11]. Symptoms of depression include persistent sadness, lack of energy, irritability, reduced capacity to concentrate, and changes in sleep and appetite lasting two or more weeks [10]. Anxiety, clinically classified as generalized anxiety disorder (GAD), includes feelings of anxiety, worry, and fear that interfere with one’s daily activities [12]. For young adult college students, anxiety is suggested to be a result of several components like worries about family and potential issues in the home, lack of confidence, social acceptance among classmates and peers, and academic performance [13].

The trauma of exposure to an active shooter and loss of life in one’s normal living environment may exacerbate these issues, [2]and other factors may help or hurt the recovery process. Low physical fitness is associated with poorer mental health outcomes [14] and is rarely considered when offering interventions [15,16]. A preponderance of evidence supports the inverse relationship between depression and fitness level [17–19]. Additionally, perceived discrimination [20,21] and sense of belonging to the university [22,23] impact mental health. Perceived discrimination has a profound association with various aspects of university students’ well-being that includes self-esteem, academic adjustment, and stress [24]. Indeed, discriminatory experiences are associated with psychological distress, anxiety, and depression [25,26]. The more students experienced perceived discrimination the lower the engagement in preventive health behaviors and higher levels of anxiety/depression and suicidal behaviors, without considering the impact of a MCE [26]. A sense of belonging is a critical measure of student well-being within a campus environment, encompassing feelings of connectedness, mattering, and being valued by the campus community, including peers, faculty, and staff. Strayhorn defines it as the “degree to which an individual feels respected, valued, accepted, and needed by a defined group” (p. 87), emphasizing its role as both a fundamental need and a motivator for student behavior [27]. Marginalization, whether due to race, cultural disconnection, or mental illness, can hinder a sense of belonging [28,29]. Less is known about how these intersecting paradigms interact with emotional recovery from a mass shooting.

Following traumatic events, such as a mass casualty event (MCE), campuses often promote counseling services to students, faculty, and staff to help mitigate mental health impacts related to the incident [2,15,30,31]. However, on-campus counseling services may be under-resourced and already in high demand due to rising rates of depression and anxiety among college students. As a result, providing long-term counseling for those affected by a mass casualty event may be infeasible. Furthermore, students are not always willing to pursue counseling and may prefer to engage in other self-care activities [15,30]. Although counseling can be an effective intervention after trauma, there remains a gap in knowledge regarding preferences for non-counseling options, such as increased physical activity or meditation.

Therefore, the purpose of this study was to examine the impact of a campus shooting resulting in mass casualties on the mental health of students and employees. In addition, the study examined whether belongingness, cardiorespiratory fitness, and perceived discrimination moderated mental health responses following the event. The study also explored preferred treatment and support options among individuals reporting symptoms of depression or anxiety. Collectively, this approach was intended to inform future institutional stakeholders by providing evidence to guide the development of effective support strategies for students and employees following such traumatic events. An anonymous survey was distributed via email, student-facing announcements, and in-class announcements with follow-up emails containing a link to the survey. The survey was distributed four to five months after the MCE. Participants were asked to retrospectively report symptoms of depression and anxiety from the period prior to the mass shooting as well as symptoms experienced during the two weeks immediately preceding survey completion. Participants were also asked to complete one questionnaire comprised of demographic questions and validated survey instruments to evaluate their current sense of belonging, perceived discrimination [32], and estimated cardiorespiratory fitness (eCRF) [33]. Finally, respondents were also asked questions regarding willingness to attend mental health counseling or learn about other wellness techniques to relieve symptoms of depression or anxiety. These factors were evaluated to better understand how to best support a campus community after a mass casualty event.

## Materials and methods

The study was exempted by the Office of Research Integrity, Social Behavioral Institutional Review Board of the University of Nevada, Las Vegas (protocol code #UNLV-2024–129, February 20, 2024). The study was classified as exempt (Category 2(i) under 45 CFR 46.104(d)(2)) as the information obtained was recorded by the investigator in such a manner that the identity of the human subjects cannot readily be ascertained, directly or through identifiers linked to the subjects. Informed consent was provided at the start of the online questionnaire, along with a trigger warning to inform participants of the inclusion of questions related to the on-campus shooting, which occurred approximately 4–5 months prior to the distribution of the questionnaire (April 12 to May 11, 2024).

Upon providing electronic informed consent, participants completed a multi-faceted, self-administered questionnaire that included several validated instruments, including the Patient Health Questionnaire-9 (PHQ-9) [34] and the Generalized Anxiety Disorder-7 (GAD-7) [35]. Notably, the PHQ-9 and GAD-7 were the only measures requiring retrospective recall. For these instruments, participants reported their mood during two time periods: (1) the two weeks immediately preceding the mass casualty event (pre-MCE recall, approximately 4–5 months prior to survey administration), and (2) the two weeks immediately preceding completion of the questionnaire (recent recall). All other measures, including the School Belongingness Scale (SBS) [36] and the Perceived Ethnic Discrimination Questionnaire (PEDQ) [32], assessed participants’ current state only.

Participants were also asked for demographic information, including university status (employee or student), grade point average (GPA; students only), age, race, ethnicity, sex, gender identity, sexual orientation, height, and weight. Body mass index (BMI) was calculated from self-reported height and weight using the formula weight (kg) / [height (m)]². A Physical Activity Index (PA-I) and estimated cardiorespiratory fitness (eCRF) were derived from participant data reported in the survey which are entered into validated algorithms for calculations (see further details below) [33,37]. Participants reported whether they were currently using antidepressant medication or engaged in counseling or psychotherapy for depression treatment. Finally, participants stated their willingness to attend counseling sessions at the university (yes/no) and their interest in learning about alternative approaches, such as meditation and exercise (yes/no).

Depressive symptom severity recall from both the 2 weeks before the MCE and for the 2 week period prior to questionnaire completion was assessed using the PHQ-9, a brief, validated self-report measure widely used for screening, monitoring, and quantifying depression severity in both research and clinical settings. The PHQ-9 consists of nine items reflecting Diagnostic and Statistical Manual of Mental Disorders, Fourth Edition (DSM-IV) criteria for major depressive disorder and assesses symptom frequency over the past two weeks. Items are scored from 0 to 3 (not at all, several days, more than half the days, or nearly every day), yielding a total score ranging from 0 to 27. Standard cut points categorize scores as follows: 0–4 (no depression), 5–9 (minimal symptoms), 10–14 (mild depression), 15–19 (moderately severe depression), and ≥20 (severe depression). A PHQ-9 score of 10 or higher demonstrates strong diagnostic accuracy for major depression, with both sensitivity and specificity of 88% [34].

Anxiety symptom severity recall, using the same time frames as the PHQ-9, was assessed using the GAD-7, a seven-question validated self-report measure widely used to screen for anxiety severity [35]. The GAD-7 consists of seven items reflecting core symptoms of generalized anxiety disorder and assesses symptom frequency over the past two weeks. Items are scored from 0 to 3 (not at all, several days, more than half the days, or nearly every day), yielding a total score ranging from 0 to 21. Standard cut points categorize scores as follows: 0–4 (minimal anxiety), 5–9 (mild anxiety), 10–14 (moderate anxiety), and ≥15 (severe anxiety). A GAD-7 score of 10 or higher has demonstrated good diagnostic accuracy for generalized anxiety disorder, with a sensitivity of 89% and specificity of 82% [35].

The School Belongingness Scale (SBS), originally developed by Goodenow (1993) as the Psychological Sense of School Membership (PSSM) Scale, was utilized in this study to assess students’ current perceptions of belonging within the university setting [36]. The SBS is a 16-item self-report measure designed to evaluate the extent to which students feel accepted, respected, and valued by their academic community. Each item is rated on a 7-point Likert scale, ranging from “strongly disagree” to “strongly agree.” The SBS has been widely used in research due to its strong psychometric properties, including high internal consistency and construct validity across diverse student populations [38]. Prior studies have linked a strong sense of school membership to higher academic achievement, increased motivation, and improved psychological well-being, while lower school belonging has been associated with feelings of isolation, anxiety, and depression [36]. The SBS was included one time in the survey and asked participants to answer the questions based on their current experience of sense of belonging.

The participant’s current perception of ethnic discrimination was assessed using the Brief PEDQ, a 17-item abbreviated form of the original 70-item PEDQ [32]. Items assess the frequency of discriminatory experiences and are rated on a 5-point Likert scale ranging from 1 (never) to 5 (very often), with higher scores indicating greater perceived discrimination. A total PEDQ score was computed by averaging responses across all 17 items. The Brief PEDQ has demonstrated acceptable to strong internal consistency, with reported Cronbach’s alpha coefficients ranging from 0.65 to 0.88 in community and student samples [32]. Consistent with the School Belongingness Scale (SBS), the PEDQ captured participants’ overall perceptions of discrimination at the time of survey completion.

Cardiorespiratory fitness was estimated using a validated and reliable algorithm to examine responses to the trauma of the mass shooting event in relation to fitness level. Cardiorespiratory fitness reflects the cumulative effect of sedentary and physical activity behaviors. The eCRF measure developed by Nes et al. has demonstrated strong validity and has been shown to be comparable in accuracy to many field-based assessments of peak oxygen consumption (VO₂peak) [33,37]. The model was derived from cross-validation VO₂peak data from 4,367 healthy adults and has demonstrated acceptable accuracy for estimating sex-specific VO₂peak in outpatient and population-based settings [33,37]. The regression model includes age, body mass index (BMI), resting heart rate (RHR), and a physical activity index (PA-I). The PA-I was calculated from self-reported exercise frequency, duration, and intensity using a previously validated scoring system [33,37,39] and has shown moderate correlations with measured VO₂peak (r = 0.44 for men; r = 0.38 for women). Although the original model incorporated waist circumference, a BMI-based algorithm has been shown to produce comparable estimates [37]. Given inconsistencies in self-reported waist circumference, the BMI-based model was used in the present study.

Sex-specific equations were applied as follows:

Men (R² = 0.59, SEE = 5.8): 92.05 − (0.327 × age) − (0.933 × BMI) − (0.167 × RHR) + (0.257 × PA-I)Women (R² = 0.57, SEE = 5.1): 70.77 − (0.244 × age) − (0.749 × BMI) − (0.107 × RHR) + (0.213 × PA-I)

For each participant, estimated CRF (eCRF) was compared with age-predicted normative CRF values for healthy adults [19,33]. To enhance methodological rigor, eCRF estimates and normative values were cross-verified using the web-based calculator developed by the original authors. Both eCRF and the difference between individual eCRF and normative CRF values (DIFF) were included in subsequent analyses.

### Statistical analysis

Given the nature of this research, there was no a priori power analysis to inform the appropriate sample size given its spontaneous and challenging conditions. Additionally, data collection was time sensitive, i.e., survey responses need to be collected over a consolidated amount of time to prevent any recall bias. However, using typical effect size standards [40] the aim was to be adequately powered to detect moderate effect sizes (r = .3) which require a total sample size of 82.

All statistical analyses were completed using R (version 4.4.1). Logistic regression was used to examine any relationship between those who completed or did not fully complete the survey, with survey completeness (yes/no) as the outcome variable and role at the university as the predictor variable. Summary statistics of those who partially complete the survey were calculated and compared to the sample carried forward to describe potential bias in partial completion.

Initial examination of differences in PHQ-9 and GAD-7 between contrasting recalls within participant (pre-MCE versus recent) were examined with Wilcoxon Signed Rank tests and differences in categorical assignment were examined with a nominal symmetry test that can handle paired contingency tables larger than 2x2. A linear mixed effects model with random intercepts for participant was used to examine individual differences in PHQ-9 with primary independent variables of recall (pre-MCE recall/recent recall), pre-MCE recall GAD-7 scores, current treatment status for anxiety or depression (yes/no), difference in actual (eCRF) to perceived (Hunt Norm) fitness, log-transformed mean PEDQ, and role (student, student and employee, and employee only). The model also included control variables of participant age, Sex (Male, Female), Sex/Gender Minority (non-SGM versus SGM), race, Latino/Hispanic ethnicity, and location at the time of the event (on-campus, near campus, off-campus). The model also examined the interaction between recall (pre-MCE versus recent) and each variable, except for age and race due to aliasing with role (i.e., role was strongly correlated with age and race). A separate but identical linear mixed effects model was used to examine differences in GAD-7 recalls as the primary outcome with pre-MCE recall PHQ-9 scores as an alternative control variable.

Sub-group analyses were conducted among respondents who reported being students, with all prior model terms remaining the same except inclusion of GPA as a covariate, SBS scores, their year at school (lower, upper, or grad), and their interaction with recall (pre-MCE versus recent) as additional independent variables.

A sub-group analysis was also performed for university employees. However, given the smaller level of response among this group, simpler models were constructed looking at main effects of recall, either pre-MCE recall GAD-7 or pre-MCE recall PHQ-9 scores, eCRF Difference, log-transformed mean PEDQ, and currently receiving treatment for mental health concerns (yes/no).

Across each model, all continuous variables were zero mean centered and scaled to their standard deviation to prevent structural collinearity in the interaction term with time. Post-hoc analyses of all statistically significant effects and interactions were examined through estimation of marginal means and trends. Chi-squared was used to examine the frequency and attitudes toward seeking treatment whether through university psychological services, and/or Exercise/Meditation.

## Results

### Participant characteristics

A total of 198 survey respondents began the survey with 157 completing it fully. Completer analysis demonstrated that those who completed the survey versus those who did not complete the survey was not related to their role at the university (χ(2) = 3.94, p = .14). Five participants did complete a portion of the survey beyond reporting of their initial role. Among these partial completers, each reported similar demographic and survey responses like those who fully completed the survey. Interestingly, each did not fully complete the PEDQ, but we have no information on why this questionnaire within the survey was left incomplete among these participants.

Respondents who fully completed the survey (N = 157) were carried forward for analysis. The mean age of respondents was 27.01 + /- 11.25, 118 (75%) female. Twenty-five (16%) respondents reported their role as employee only, 116 (74%) reporting as student only, and 16 (10%) reporting student and employee status. Complete description of demographic and other survey responses can be viewed in Table 1.

**Table 1:** Aggregate participant demographics and survey responses.

	(n = 157)
**Participant Demographics**	
**Characteristic**	**Value**
**Age**	27.01 (11.25)
**Assigned Sex**	
Female	118 (75%)
Male	39 (25%)
**Race**	
Asian	32 (20%)
Black/African American	5 (3.2%)
Latino	34 (22%)
More than one race	26 (17%)
Native Alaskan/American Indian	3 (1.9%)
No Race Reported	6 (3.8%)
White	51 (32%)
**Hispanic/Latino Ethnicity**	
Hispanic or Latino/Latina	53 (34%)
Not Hispanic or Latino/Latina	102 (66%)
Unknown	2
**Sexual Minority**	
Sexual Gender Minority	30 (19%)
Straight	127 (81%)
**Currently receiving treatment for depression or anxiety (medication, counseling, or psychotherapy)**	
Yes	27 (17%)
No	130 (83%)
**Role**	
A student and employee	16 (10%)
A student	116 (74%)
An employee	25 (16%)
**Survey Responses**	
**Shooting Location**	
Off campus.	71 (45%)
On campus in another area.	56 (36%)
On campus near the Student Union.	30 (19%)
**Perceived Discrimination (PEDQ)**	1.35 (0.49)
**School Belongingness Scale Total (SBS)**	83.29 (19.41)
Incomplete^a^	25
**Estimated Cardiorespiratory Fitness(eCRF)**	43.92 (8.60)
**Difference between eCRF & age predicted CRF**	−1.32 (6.17)

Mean (SD); n (%).

^a^Employees did not complete the SBS questionnaire.

#### Differences in pre-MCE and recent recall on the PHQ-9 and GAD-7.

**Fig 1:**
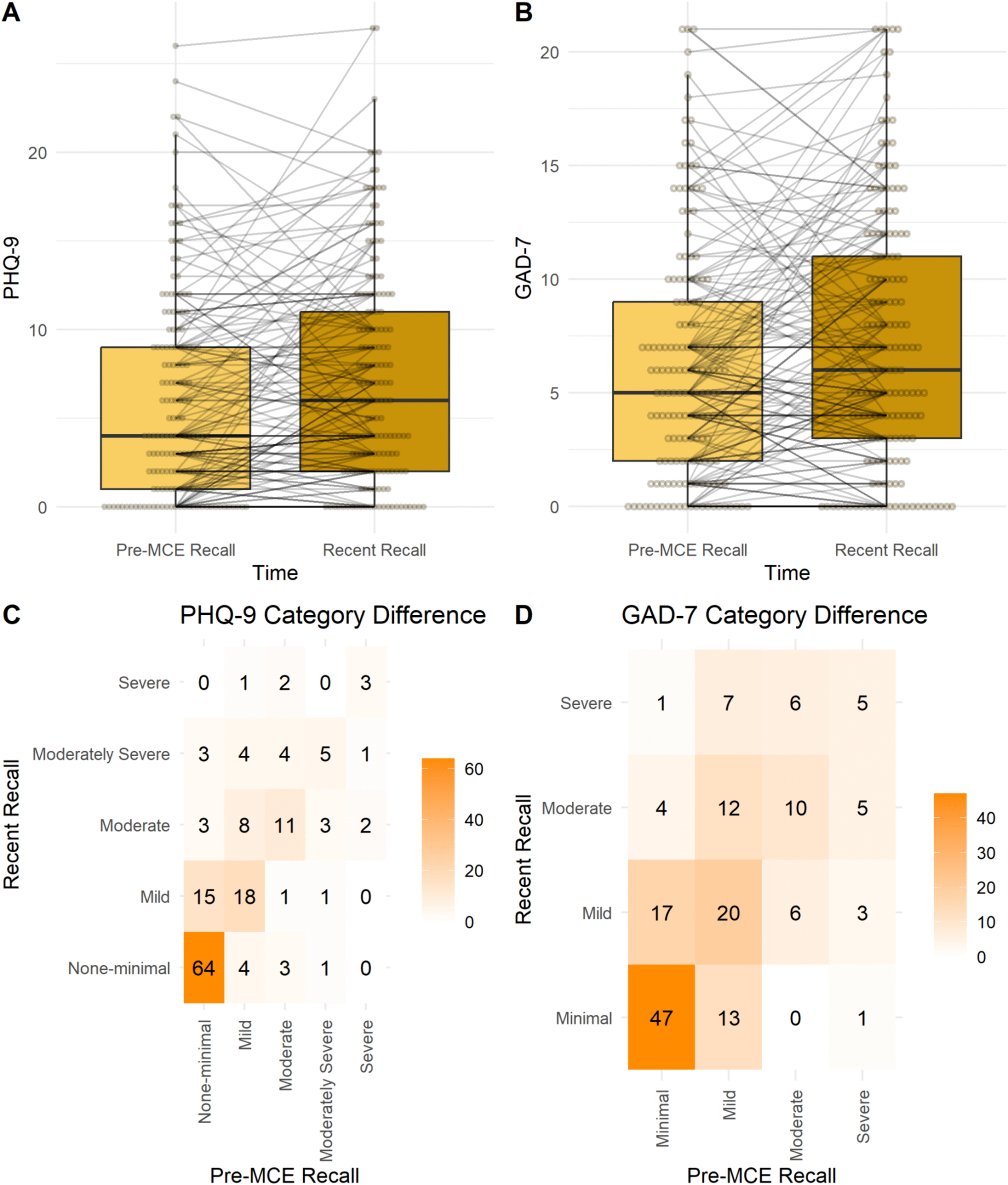
Recall Difference in PHQ-9 and GAD-7. Boxplots for (A) PHQ-9 and (B) GAD-7 scores as reported by participants of their pre-MCE recall scores compared to their recent recall scores. Heatmap of categorized differences of PHQ-9 (C) and GAD-7 (D) scores with pre-MCE recall calculated categories on the x-axis and recent recall calculated categories on the y-axis.

**Fig 2:**
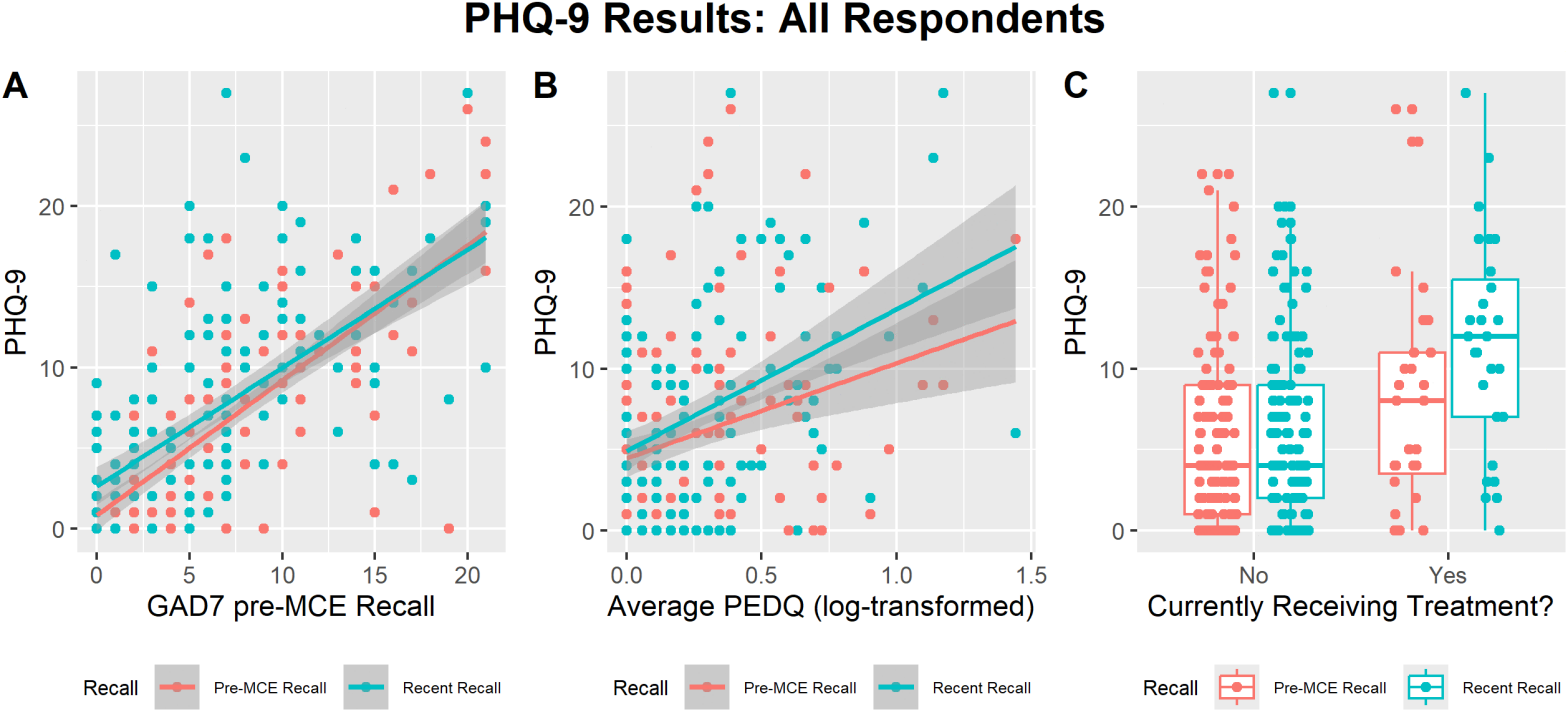
PHQ-9 Results: All Respondents. Differences in PHQ-recall scores (pink = pre-MCE recall, blue = recent recall) among all respondents as a function of (A) pre-MCE recall GAD-7 scores, (B) mean PEDQ (log-transformed) and (C) currently receiving treatment for anxiety and/or depression.

Results of the Wilcoxon Signed Rank test for differences in PHQ-9 and GAD-7 recall were statistically significant with an increase from pre-MCE recall to recent recall (PHQ-9 difference = 1.18, p = .001, Fig 1A, and GAD-7 difference = 1.04, p = .005, Fig 1B). Summary statistics reported in Table 2. There was no statistically significant difference in individual assessment categorization between recall types (pre-MCE recall versus recent recall) for PHQ-9 (Fig 1C) or GAD-7 (Fig 1D) based on results from Mcnemar tests. Visualization of assessment categorization can be viewed in Fig 2.

**Table 2:** Depression (PHQ-9) and anxiety (GAD-7) scores pre-MCE recall and recent recall.

Characteristic	Pre-MCE RecallN = 157	Recent RecallN = 157	p-value
**PHQ-9**	5.96 (5.87)	7.13 (6.19)	**0.001**
**GAD-7**	6.12 (5.23)	7.16 (5.76)	**0.005**

Mean (SD); Wilcoxon signed rank test with continuity correction; PHQ-9, Patient Health Questionnaire-9 questions; GAD-7, Generalized Anxiety Disorder-7-questions.

#### Effects and Modifiers of MCE on pre-MCE and recent recall on PHQ-9 scores (All Respondents).

Results of the linear mixed effects model that examined differences in recalled PHQ-9 scores (pre-MCE recall/recent recall) demonstrated no main effect of recall (β_RecentRecall_ = −0.38, 95% CI = [−3.48; 2.72], p = 0.82). There was a significant relationship between GAD-7 pre-MCE recall scores and PHQ-9 across both recall time points (β_GAD-7(pre-MCErecall)_ = 4.16, 95% CI = [3.43; 4.89], p < .001). This result indicates that respondents who reported higher pre-MCE GAD-7 scores also reported higher PHQ-9 scores for each recall. The interaction between recall period and pre-MCE recall GAD-7 score was significant (β = −1.09, 95% CI = [−1.83, −0.35], p = .006, Fig 2A): participants with lower pre-MCE recall GAD-7 scores showed the largest increases in recent recall PHQ-9, while participants with higher pre-MCE recall GAD-7 scores showed little change. There was also a significant recall by log-transformed mean PEDQ interaction (β_RecentRecall_:_PEDQ_ = 3.03, 95% CI = [0.41; 5.67], p = .03), which indicates that individuals who reported less discrimination reported a smaller difference in their PHQ-9 scores than individuals who reported more perceived discrimination (Fig 2B). Furthermore, there was also an anxiety and depression by recall interaction (β_RecentRecall_:_Treatment(Yes)_ = 3.3, 95% CI = [1.51; 5.18], p < .001, Fig 2C), where respondents who reported currently receiving treatment for anxiety and/or depression reported a 3.3 point relative increase in their PHQ-9 score compared to those not currently receiving treatment. Examination of estimated marginal means among those who reported not receiving treatment did not demonstrate a significant difference in their PHQ-9 pre-MCE recall/recent recall scores (No Treatment: pre-MCE recall – recent recall = 0.60, p = .36), compared to those that do receive treatment (Yes Treatment: pre-MCE recall – recent recall = 3.95, p = < .001). No effect of cardiorespiratory fitness was observed.

#### Effects and Modifiers of MCE on pre-MCE and recent recall on PHQ-9 scores (Students Only).

**Fig 3:**
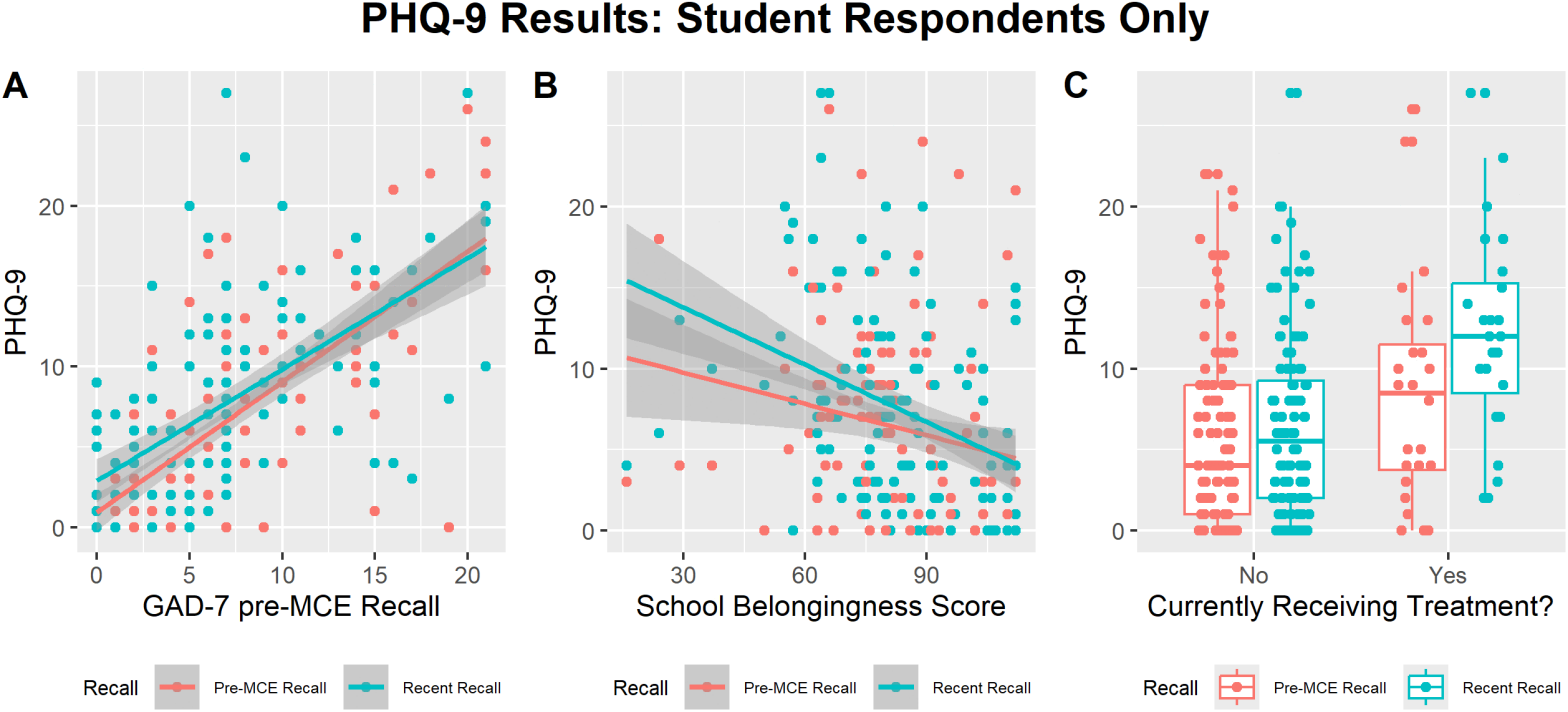
PHQ-9 Results: Students Only. Difference in PHQ-9 due to event as a function of pre-GAD-7 scores (A), Total SBS scores (B) and currently receiving treatment for anxiety and/or depression (C) among student respondents only.

Sub-group analysis among student respondents demonstrated a significant relationship between pre-MCE recall GAD-7 scores and PHQ-9 (β_GAD-7(pre-MCErecall)_ = 3.92, 95% CI = [3.17; 4.68], p < .001) and an interaction between recall and pre-MCE recall GAD-7 scores (β_RecentRecall:GAD-7(pre-MCErecall)_ = −1.2, 95% CI = [−1.93; −.40], p = .004). This result is consistent with the all respondent model and indicates that students GAD-7 scores are related to PHQ-9 scores and students with lower pre-MCE recall GAD-7 scores experienced greater increases in recent recall PHQ-9 (Fig 3A). The interaction between recall and log mean PEDQ was no longer significant (β_RecentRecall:PEDQ_ = 1.5, 95% CI = [−1.72; 4.05], p = .45). However, a significant interaction between recall and SBS scores emerged (β_RecentRecall:SBS_ = −0.87, 95% CI = [−1.66; −0.08], p = .04, Fig 3B), which indicates that student respondents who reported lower belongingness experienced a greater increase in PHQ-9 scores. There was only a trend of GPA to belongingness, whereby students with lower GPAs had lower belongingness scores. The interaction between recall and treatment was also statistically significant among student respondents (β_RecentRecall:Treatment(Yes)_ = 3.4, 95% CI = [1.45; 5.33], p = .001, Fig 3C). No effect of cardiorespiratory fitness was observed.

#### Effects of MCE on pre-MCE and recent recall on PHQ-9 scores (Employees Only).

Sub-group analysis among employees utilized a simpler model to avoid overfitting that included main effects of Recall (pre-MCE versus recent), GAD-7 scores (pre-MCE recall), eCRF Difference, average PEDQ (log-transformed), and current treatment (yes/no). Only GAD-7 scores (pre-MCE recall) were related to PHQ-9 scores among employees (β_GAD-7(pre-MCErecall)_ = 4.44, 95% CI = [3.12; 5.77], p < .001). On average employees had lower average PHQ-9 scores across the combined recalls than students (employee = 4.6; student = 6.9), they both reported roughly the same difference from pre-MCE recall to recent recall (PHQ-9 employee recall difference = 1.2, PHQ-9 student recall difference = 1.17). No effect of cardiorespiratory fitness was observed.

#### Effects of MCE on pre-MCE and recent recall on GAD-7 scores (All Respondents).

**Fig 4:**
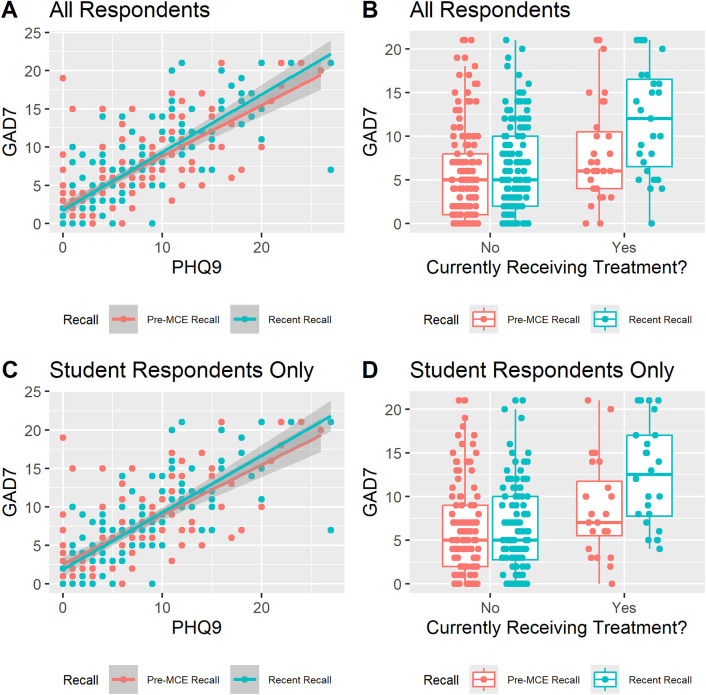
GAD-7 Results: All Respondents and Students Only. The relationship between pre-PHQ-9 scores and GAD-7 pre and post MCE among all respondents (A) and student respondents only (C). Differences in GAD-7 scores between those who are and who are not currently receiving treatment for anxiety or depression among all respondents (B) and student respondents only (D).

Results of the linear mixed effects model among all respondents that examined differences in recalled GAD-7 scores from pre-MCE to recent recall demonstrated no main effect of event (β_RecentRecall_ = 1.43, 95% CI = [−1.64; 4.51], p = .38). However, there was a significant effect of pre-MCE recall PHQ-9 scores (β_PHQ-9(pre-MCErecall)_ = 3.52, 95% CI = [2.89; 4.15], p < .001, Fig 4A), which indicates that individuals who reported higher PHQ-9 had higher GAD-7 scores than those who reported lower scores. There was also a significant relationship with log mean PEDQ (β_PEDQ_ = 2.52, 95% CI = [.28; 4.75], p = .04). Furthermore, there was a significant recall by treatment interaction (β_RecentRecall:Treatment(Yes)_ = 3.27, 95% CI = [1.46; 5.09], p = .001, Fig 4B), which indicates that respondents who reported currently receiving treatment for anxiety or depression experienced a 3.27 relative increase in their GAD-7 event-based recalled scores compared to those who are not currently receiving treatment for anxiety or depression. Consistent with the PHQ-9 results, respondents who reported not receiving treatment did not demonstrate a significant difference in their GAD-7 (p = .15). No effect of cardiorespiratory fitness was observed.

#### Effects of MCE on pre-MCE and recent recall on GAD-7 scores (Students Only).

Sub-group analysis among student respondents were consistent with that of the all-respondent analysis with a significant relationship between pre-MCE recall PHQ-9 scores (β_PHQ-9(pre-MCErecall)_ = 3.46, 95% CI = [2.77; 4.17], p < .001, Fig 4C) and a trend with log-transformed, average PEDQ scores (β_PEDQ_ = 2.58, 95% CI = [0.18; 4.98], p = .05). A similar recall by treatment interaction was also observed (β_RecentRecall:Treatment(Yes)_ = 3.56, 95% CI = [1.66; 5.47], p < .001, Fig 4D). No effect of cardiorespiratory fitness was observed.

#### Effects of MCE on pre-MCE and recent recall on GAD-7 scores (Employees Only).

Sub-group analysis among employees utilized a simpler model with only main effects of Recall (pre-MCE recall versus recent recall), GAD-7 scores (pre-MCE recall), eCRF Difference, average PEDQ (log-transformed), and current treatment (yes/no). Results demonstrated a main effect of Recall (β_RecentRecall_ = 2.36, 95% CI = [0.68; 3.44], p = .01), which indicates an average difference of 2.36 points in GAD-7 from pre-MCE recall to recent recall. There was also a statistically significant relationship with pre-MCE recall PHQ-9 scores (β_PHQ-9(pre-MCErecall)_ = 3.47, 95% CI = [2.55; 4.36], p < .001), where greater pre-MCE recall PHQ-9 scores were positively related to GAD-7 scores. There was also a significant relationship between log-transformed PEDQ scores and PHQ-9 (β_PEDQ_ = 4.92, 95% CI = [1.37; 8.47], p = .02), which indicates that employees who reported higher perceived discrimination also had higher anxiety (GAD-7 scores). No effect of cardiorespiratory fitness was observed.

#### Frequency and Attitudes toward Seeking Help Either through University Psychological Services or Alternative Methods.

Exploratory analysis of treatment seeking and attitudes toward treatment post event demonstrated that of the 157 total respondents, 27 (17%) were currently receiving treatment (antidepressant medication, counseling, or psychotherapy) for depression or anxiety. Of these 27, 2 (1%) reported initiating treatment because of the event. Each of these respondents receiving treatment for the event were students. Among all respondents, when asked if they would seek university mental health services if they felt anxious or depressed, 52 (34%) replied they would not, and 105 (66%) responded in the affirmative.

Respondents who reported they would not seek university mental health services reported a variety of reasons including: already having a primary doctor or seeking service outside of school, feeling shame for seeking service, feeling their anxiety does not meet the need for treatment, quality of university services, and handling it on their own or with family.

**Fig 5:**
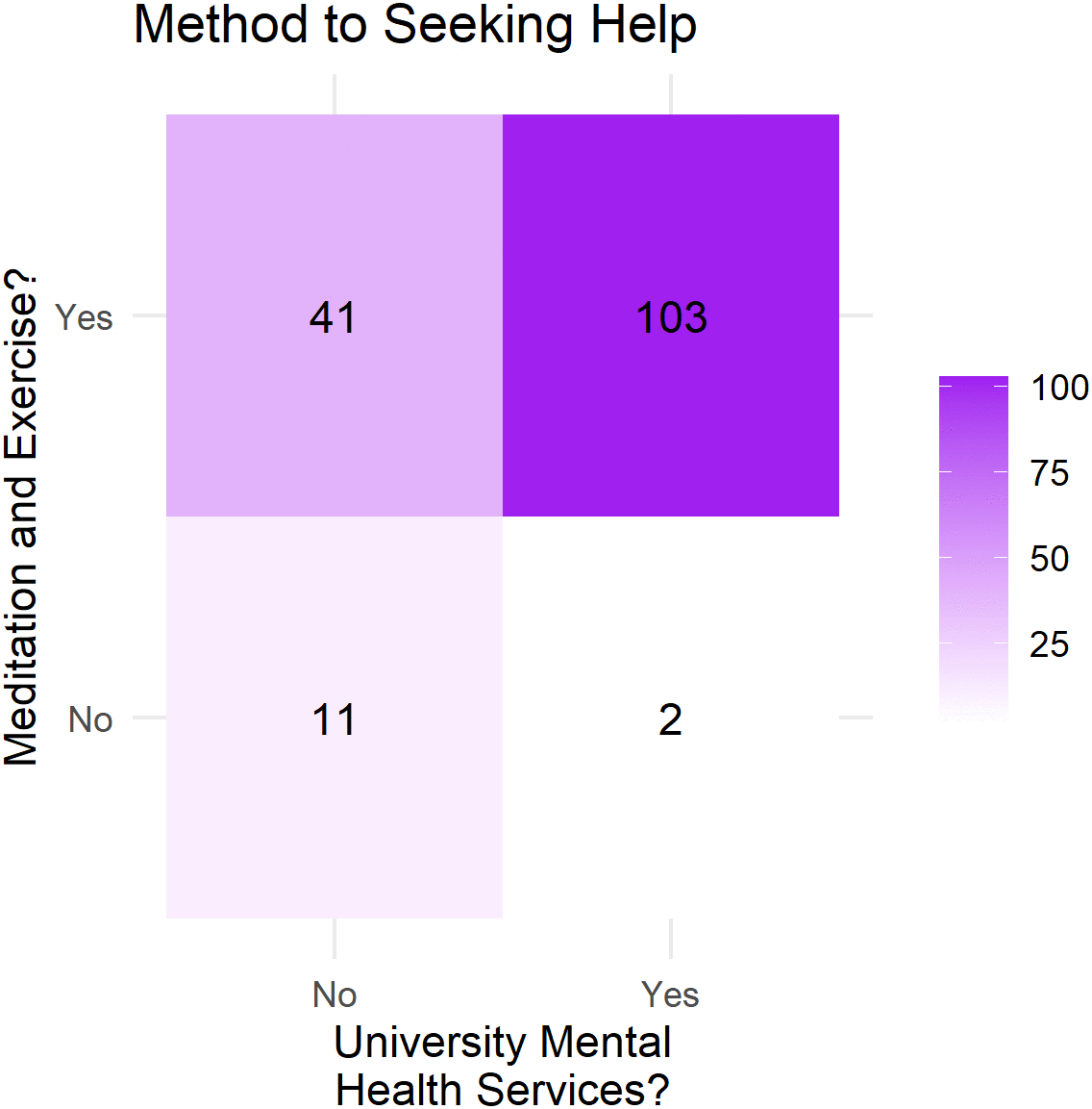
Preferred Method for Seeking Help. Heatmap demonstrating the difference in proportion of respondents who would seek meditation and exercise over university counseling services.

When all respondents were asked if they would seek alternative treatment, such as exercise and/or meditation, if they felt anxious or depressed, 13 (8%) replied they would not and 144 (92%) responded in the affirmative. A chi-square test that examined the difference in proportion between individuals who would seek university mental health services versus exercise/meditation for treatment of anxiety or depression was statistically significant (χ2 = 14.527, df = 1, p < .001, Fig 5). This result indicates a greater preference among respondents to seek treatment due to depression and anxiety through exercise and meditation rather than university mental health services.

Respondents who reported they would not seek alternative treatment (N = 13, 8%) if they felt anxious or depressed reported various reasons including: such treatments would not work, they don’t have time, they are not depressed/anxious, they feel a need to just move on

## Discussion

This study sought to understand the mental health impact of a campus mass-casualty shooting on students and employees, as well as the potential moderating roles of belongingness, cardiorespiratory fitness, and perceived discrimination in post-event psychological responses. The study also explored treatment and support preferences among individuals reporting symptoms of depression or anxiety. The results indicated a significant perceived increase in self-reported depression and anxiety from before the MCE to the time of the survey completion. However, the magnitude of the difference was dependent on the level of anxiety and depression experienced before the shooting, perceived discrimination, and participation in treatment for anxiety and/or depression. Specifically, among students’ perceived sense of school belongingness also modified individual depression, while among employees perceived discrimination was significantly to their anxiety. Indeed, a key outcome was the interaction between current treatment for anxiety or depression and mental health outcomes. Those already engaged in mental health treatment before the MCE exhibited greater increases in PHQ-9 and GAD-7 scores than their untreated peers. This suggests that individuals with pre-existing mental health conditions may be particularly vulnerable to exacerbations following traumatic events. It is interesting to note that the greatest proportion of those who reported currently receiving treatment were non-Hispanic, White females.

Those who reported higher levels of perceived discrimination exhibited greater increases in depressive symptoms, corroborating previous research demonstrating the adverse effects of marginalization on mental health [24,26]. Similarly, sense of belonging emerged as a trending protective factor, with lower belongingness scores predicting more significant increases in depression. These results align with existing literature emphasizing the importance of fostering inclusive campus environments to mitigate the negative psychological consequences of discrimination [27,41].

The preference for alternative coping mechanisms over formal mental health services is noteworthy. A key component to recovery should be to provide opportunities to students to help themselves not only survive a traumatic event, but thrive afterward [42]. Thus, other interventions, such as referrals to faith-based organizations [43], increased physical activity or meditation have been shown to reduce symptoms of depression and anxiety and could be considered as additional or alternative resources. Resiliency can be fostered through any number of resources, and the ability to foster one’s own resilience has been shown to improve coping ability after traumatic events such as campus shootings [42,43]. A substantial proportion of respondents indicated a willingness to engage in exercise or meditation to manage symptoms of anxiety and depression, while fewer were inclined to seek university counseling services. This finding underscores the necessity for universities to broaden their mental health support strategies beyond traditional counseling, incorporating wellness-based interventions that align with preferences and accessibility [15]. Given the increasing prevalence of mental health concerns among college students, a holistic approach to psychological support, encompassing traditional therapy, peer support networks, and wellness initiatives, may be most effective in fostering recovery.

In this questionnaire, respondents reported a key barrier to seeking counseling services on campus were due to logistical difficulties, including scheduling constraints, long wait times, and perceived inefficacy of university mental health services. Many students expressed concerns about the compatibility of services with their needs, and some were deterred by the stigma associated with seeking professional help. Additionally, participants who had pre-existing mental health care providers outside the university often preferred to continue with their established therapists rather than transitioning to university-based counseling. These findings suggest that universities need to enhance the accessibility and perceived quality of their mental health services to better support those in need.

Alternatively, participants demonstrated a higher willingness to pursue alternative interventions such as exercise and meditation. Despite this preference, some cited barriers to engaging in these methods, including time constraints, lack of motivation, or physical limitations such as injuries. While alternative therapies may not be suitable for everyone, their popularity highlights the need for universities to promote holistic wellness strategies that integrate mental health and physical activity. Expanding access to structured wellness programs, peer-led initiatives, and educational campaigns on the mental health benefits of exercise and meditation could encourage broader participation and improve student well-being.

Although the results of this study did not include significant associations between fitness and other variables, a substantial body of evidence indicates that improved cardiorespiratory fitness, a result of regular physical activity, improves mental health [44–46]. Previous research suggests that low fitness is linked to increased depressive symptoms in college students [19], and exercise is shown to be a crucial factor in maintaining their mental and cognitive well-being [47–49]. The relationship between mental health and exercise as a behavior or fitness as a long-term outcome underscores the potential for targeted interventions that incorporate fitness assessments and physical activity as preventive strategies. Given the accessibility of exercise-based programs on college campuses, integrating structured fitness initiatives may serve as a viable approach to improving mental health resilience in university populations.

Another consideration is mood-congruent memory, where prior research suggests that individuals may preferentially recall information consistent with their current emotional state, which could influence how participants reconstruct past experiences [50,51]. This may have affected participants’ pre-MCE recall, particularly among those reporting higher anxiety or depression at survey completion. However, prior research examining anxiety and depression among university students suggests that pre-MCE recall scores observed in our sample are comparable to those reported in the literature [52]. This comparability supports that the recall differences observed here are valid and suggests that participants’ retrospective reports were less influenced by mood-based bias than expected. Specifically, if participants were systematically inflating the positivity of their pre-MCE experiences, we would expect lower levels of anxiety and depression than typically reported, which we did not observe. Conversely, if respondents were normalizing or minimizing distress from the MCE, we would expect a smaller difference between pre-MCE and recent recall reports. Together, these patterns suggest that mood-congruent memory may not substantially explain the recall differences observed here, though this interpretation warrants caution.

### Limitations

Several limitations of this study should be acknowledged. First, the study relied on self-reported measures of anxiety, depression, and fitness levels, which may be subject to response bias and inaccuracies in self-assessment. Future research could benefit from incorporating objective measures such as clinical assessments and physiological indicators to provide a more comprehensive understanding of mental health outcomes. Second, the incorporation of objective measures poses a challenge in the context of MCEs due to their rarity and unpredictability. While subjective measures may not represent the gold standard in terms of construct validity, they are likely the most feasible and commonly accepted approach in this area of research. Third, the study design was cross-sectional and based on a relatively small number of respondents, limiting the ability to establish causal relationships between variables. Longitudinal studies are needed to explore how anxiety, depression, and fitness levels evolve over time following mass casualty events and whether specific interventions have sustained effects on mental health. Fourth, the sample was drawn from a single minority-serving institution, which may limit the generalizability of the findings to other student populations with different demographic compositions and institutional resources. Expanding research to multiple universities with diverse student populations could enhance the external validity of the results. Fifth, mood-congruent memory theory suggests that recall is biased toward information consistent with an individual’s current emotional state [50,51]. Accordingly, participants with elevated depressive or anxiety symptoms at assessment may have preferentially recalled mood-congruent experiences from the pre-MCE recall period, potentially inflating self-reported depression and anxiety. The use of validated instruments (PHQ-9, GAD-7) may help mitigate, but not eliminate, this limitation. Additionally, pre-MCE recall scores in our sample are comparable to those reported in prior studies of university students [52]. Finally, while the study examined perceived discrimination, sense of belonging, and fitness levels as potential moderators of mental health outcomes, other influential factors such as social support, coping strategies, and personality traits were not included in the analysis. Future research should incorporate a broader range of psychological and environmental factors to better understand the complex interplay between individual and contextual influences on student mental health.

## Conclusion

Overall, these findings highlight the complex interplay between trauma, mental health, and campus climate. These findings are also subject to recall bias, particularly mood’s influence on retrospective reporting of pre-MCE symptoms. Beyond offering opportunities for mental health counseling, institutional efforts to promote belongingness, address discrimination, and expand mental health resources are critical in mitigating the psychological consequences of a MCE. Future research could explore longitudinal outcomes to determine the long-term effectiveness of different interventions and assess how various student subgroups may require tailored support strategies. By adopting a holistic approach to student well-being, universities can better equip their community to cope with trauma and foster a resilient campus community.

## References

[1] Welding L. Shootings at Colleges: U.S. Statistics. (accessed on January 5). Available from: https://www.bestcolleges.com/research/college-shootings-statistics/

[2] Peterson JK, Densley JA, Hauf M, Moldenhauer J. Epidemiology of Mass Shootings in the United States. Annu Rev Clin Psychol. 2024;20(1):125–48. doi: 10.1146/annurev-clinpsy-081122-010256 38346290

[3] Investigative Assistance for Violent Crimes Act of 2012. 6&nbsp;U.S.C. 455. 2012.

[4] Wolfe EB, Prokupecz S, Park H, Yan H. What we know about the school shooting that left 2 people dead at a private Christian school in Madison, Wisconsin. Accessed 2024 January 5. Available from: https://www.cnn.com/2024/12/16/us/madison-wisconsin-school-shooting/index.html#:~:text=The%20attack%20at%20Abundant%20Life%20is%20at,and%2027%20on%20university%20and%20college%20campuses

[5] Cimolai V, Schmitz J, Sood AB. Effects of Mass Shootings on the Mental Health of Children and Adolescents. Curr Psychiatry Rep. 2021;23(3):12. doi: 10.1007/s11920-021-01222-2 33570688

[6] Rapa LJ, Katsiyannis A, Scott SN, Durham O. School Shootings in the United States: 1997-2022. Pediatrics. 2024;153. doi: 10.1542/peds.2023-064311 38433681

[7] Ibrahim AK, Kelly SJ, Adams CE, Glazebrook C. A systematic review of studies of depression prevalence in university students. J Psychiatr Res. 2013;47(3):391–400. doi: 10.1016/j.jpsychires.2012.11.015 23260171

[8] Asher BlackDeer Msw PhD Candidate A, Patterson Silver Wolf PhD DA, Maguin PhD E, Beeler-Stinn PhD S. Depression and anxiety among college students: Understanding the impact on grade average and differences in gender and ethnicity. J Am Coll Health. 2023;71(4):1091–102. doi: 10.1080/07448481.2021.1920954 34242525

[9] Eisenberg D, Ketchen Lipson S, Heinze J, Zhou S. The Healthy Minds Study 2023-2024. University of Michigan School of Public Health; 2024. p. 1–26.

[10] NIMH. Major Depression. (accessed on January 20). Available online: https://www.nimh.nih.gov/health/statistics/major-depression

[11] Eisenberg D, Gollust SE, Golberstein E, Hefner JL. Prevalence and correlates of depression, anxiety, and suicidality among university students. Am J Orthopsychiatry. 2007;77(4):534–42. doi: 10.1037/0002-9432.77.4.534 18194033

[12] Dobson KS. An analysis of anxiety and depression scales. J Pers Assess. 1985;49(5):522–7. doi: 10.1207/s15327752jpa4905_10 4067800

[13] Barlow DH. ProQuest. Clinical handbook of psychological disorders: a step-by-step treatment manual, Sixth edition. New York: The Guilford Press; 2021.

[14] Coughenour C, Gakh M, Pharr JR, Bungum T, Jalene S. Changes in Depression and Physical Activity Among College Students on a Diverse Campus After a COVID-19 Stay-at-Home Order. J Community Health. 2021;46(4):758–66. doi: 10.1007/s10900-020-00918-5 33165765 PMC7649574

[15] Jalene S, Pharr J, Sharma M, Poston B. Depression, fitness, and student willingness to pursue university counseling and alternative antidepressant options. J Educ Health Promot. 2021;10:480. doi: 10.4103/jehp.jehp_1421_20 35233427 PMC8826772

[16] Winzer R, Lindberg L, Guldbrandsson K, Sidorchuk A. Effects of mental health interventions for students in higher education are sustainable over time: a systematic review and meta-analysis of randomized controlled trials. PeerJ. 2018;6:e4598. doi: 10.7717/peerj.4598 29629247 PMC5885977

[17] Noetel M, Sanders T, Gallardo-Gómez D, Taylor P, Del Pozo Cruz B, van den Hoek D, et al. Effect of exercise for depression: systematic review and network meta-analysis of randomised controlled trials. BMJ. 2024;384:e075847. doi: 10.1136/bmj-2023-075847 38355154 PMC10870815

[18] Malagodi F, Findon JL, Gardner B, Dommett EJ. A systematic review of the effectiveness of physical activity interventions for improving mental health and wellbeing in university students. J Coll Stud Ment Health. 2025;1–37. doi: 10.1080/28367138.2025.2566914

[19] Jalene S, Pharr J, Shan G, Poston B. Estimated Cardiorespiratory Fitness Is Associated With Reported Depression in College Students. Front Physiol. 2019;10:1191. doi: 10.3389/fphys.2019.01191 31620016 PMC6759774

[20] Bravo AJ, Wedell E, Villarosa-Hurlocker MC, Looby A, Dickter CL, Schepis TS, et al. Perceived racial/ethnic discrimination among young adult college students: Prevalence rates and associations with mental health. J Am Coll Health. 2023;71(7):2062–73. doi: 10.1080/07448481.2021.1954012 34398695 PMC8847537

[21] Lowe SR, Tineo P, Young MN. Perceived discrimination and major depression and generalized anxiety symptoms: In Muslim American college students. J Relig Health. 2019;58:1136–45. doi: 10.1007/s10943-018-0684-1 30094677

[22] Robert WM, Martin S, Virginia P. Emotional Intelligence, Belongingness, and Mental Health in College Students. Front Psychol. 2020. doi: 10.3389/fpsyg.2020.00093 PMC7006433 32076414

[23] Dutcher JM, Lederman J, Jain M, Price S, Kumar A, Villalba DK, et al. Lack of Belonging Predicts Depressive Symptomatology in College Students. Psychol Sci. 2022;33(7):1048–67. doi: 10.1177/09567976211073135 35735353 PMC13171050

[24] Hu Y, Purol SM, Shen Y, Zheng Y. Perceived racism and well-being in university racial/ethnic minority students: the complex roles of racial/ethnic identity and self-esteem. Curr Psychol. 2024;43:37196–207. doi: 10.1007/s12144-024-07099-7

[25] Hwang W-C, Goto S. The impact of perceived racial discrimination on the mental health of Asian American and Latino college students. Cult Divers Ethn Min Psychol. 2008;14(4):326–35. doi: 10.1037/1099-9809.14.4.326 18954168

[26] Morton SCM, Everhart R, Dautovich N, Chukmaitov A. Perceived discrimination and mental health outcomes in college students: the mediating effect of preventive health behaviors and social support. J Am Coll Health. 2025;73(6):2380–9. doi: 10.1080/07448481.2023.2286462 38010405

[27] Strayhorn TL. College students’ sense of belonging: A key to educational success for all students. 2nd ed. New York (NY): Routledge; 2019.

[28] Salzer MS. A comparative study of campus experiences of college students with mental illnesses versus a general college sample. J Am Coll Health. 2012;60(1):1–7. doi: 10.1080/07448481.2011.552537 22171723

[29] Hale CJ, Hannum JW, Espelage DL. Social support and physical health: the importance of belonging. J Am Coll Health. 2005;53(6):276–84. doi: 10.3200/JACH.53.6.276-284 15900991

[30] D’Amico N, Mechling B, Kemppainen J, Ahern NR, Lee J. American college students’ views of depression and utilization of on-campus counseling services. J Am Psychiatr Nurses Assoc. 2016;22:302–11. doi: 10.1177/1078390316648777 27220991

[31] Downs MF, Eisenberg D. Help seeking and treatment use among suicidal college students. J Am Coll Health. 2012;60(2):104–14. doi: 10.1080/07448481.2011.619611 22316407

[32] Brondolo E, Kelly KP, Coakley V, Gordon T, Thompson S, Levy E, et al. The Perceived Ethnic Discrimination Questionnaire: Development and Preliminary Validation of a Community Version. J Appl Soc Psychol. 2005;35:335–65. doi: 10.1111/j.1559-1816.2005.tb02124.x

[33] Nes BM, Janszky I, Vatten LJ, Nilsen TIL, Aspenes ST, Wisløff U. Estimating V·O 2peak from a nonexercise prediction model: the HUNT Study, Norway. Med Sci Sports Exerc. 2011;43(11):2024–30. doi: 10.1249/MSS.0b013e31821d3f6f 21502897

[34] Spitzer RL, Kroenke K, Williams JW, Patient Health Questionnaire Primary Care Study G. Validation and utility of a self-report version of prime-md: The phq primary care study. JAMA. 1999;282(18):1737–44. doi: 10.1001/jama.282.18.1737 10568646

[35] Spitzer RL, Kroenke K, Williams JBW, Löwe B. A brief measure for assessing generalized anxiety disorder: the GAD-7. Arch Intern Med. 2006;166(10):1092–7. doi: 10.1001/archinte.166.10.1092 16717171

[36] Goodenow C. The psychological sense of school membership among adolescents: Scale development and educational correlates. Psychol Schools. 1993;30(1):79–90. doi: 10.1002/1520-6807(199301)30:1<79::AID-PITS2310300113>3.0.CO2-X

[37] Nes BM, Vatten LJ, Nauman J, Janszky I, Wisløff U. A simple nonexercise model of cardiorespiratory fitness predicts long-term mortality. Med Sci Sports Exerc. 2014;46(6):1159–65. doi: 10.1249/MSS.0000000000000219 24576863

[38] Arslan G, Duru E. Initial development and validation of the school belongingness scale. Child Ind Res. 2017;10(4):1043–58. doi: 10.1007/s12187-016-9414-y

[39] Nauman J, Nes BM, Lavie CJ, Jackson AS, Sui X, Coombes JS, et al. Prediction of Cardiovascular Mortality by Estimated Cardiorespiratory Fitness Independent of Traditional Risk Factors: The HUNT Study. Mayo Clin Proc. 2017;92(2):218–27. doi: 10.1016/j.mayocp.2016.10.007 27866655

[40] Sullivan GM, Feinn R. Using Effect Size-or Why the P Value Is Not Enough. J Grad Med Educ. 2012;4(3):279–82. doi: 10.4300/JGME-D-12-00156.1 23997866 PMC3444174

[41] Johnson KE, Taliaferro LA. Relationships between physical activity and depressive symptoms among middle and older adolescents: a review of the research literature. J Spec Pediatr Nurs. 2011;16(4):235–51. doi: 10.1111/j.1744-6155.2011.00301.x 21951351

[42] Nugent NR, Sumner JA, Amstadter AB. Resilience after trauma: from surviving to thriving. Eur J Psychotraumatol. 2014;5:10.3402/ejpt.v5.25339. doi: 10.3402/ejpt.v5.25339 25317260 PMC4185140

[43] Brymer M, Layne C, Pynoos R, Ruzek J, Steinberg A, Vernberg E, et al. Psychological First Aid: Field Operations Guide. 2nd ed. 2006.

[44] Gao R, Wang H, Liu S, Wang X, Song S, Wang Y. Study on anxiety, depression, and sleep conditions and their interrelations among vocational college students during the COVID-19 pandemic management normalization. Front Public Health. 2024;12:1385639. doi: 10.3389/fpubh.2024.1385639 39583071 PMC11581966

[45] Guo S, Liu F, Shen J, Wei M, Yang Y. Comparative efficacy of seven exercise interventions for symptoms of depression in college students: A network of meta-analysis. Medicine (Baltimore). 2020;99(47):e23058. doi: 10.1097/MD.0000000000023058 33217806 PMC7676569

[46] Schuch FB, Vancampfort D, Richards J, Rosenbaum S, Ward PB, Stubbs B. Exercise as a treatment for depression: A meta-analysis adjusting for publication bias. J Psychiatr Res. 2016;77:42–51. doi: 10.1016/j.jpsychires.2016.02.023 26978184

[47] Huang Z, Chen B, Dong X, He J, Liu Y, Li J, et al. Association between 24-hour movement behavior and depression in college students: A compositional data analysis. J Affect Disord. 2025;369:531–7. doi: 10.1016/j.jad.2024.10.039 39395676

[48] Zhang H, Hashim SB, Huang D, Zhang B. The effect of physical exercise on depression among college students: a systematic review and meta-analysis. PeerJ. 2024;12:e18111. doi: 10.7717/peerj.18111 39329135 PMC11426321

[49] He X. Physical activity in the treatment of depression in college students. Rev Bras Med Esporte. 2022;28(1):68–71. doi: 10.1590/1517-8692202228012021_0489

[50] Faul L, LaBar KS. Mood-congruent memory revisited. Psychol Rev. 2023;130:1421–56. doi: 10.1037/rev0000394 36201828 PMC10076454

[51] Matt GE, Vázquez C, Campbell WK. Mood-congruent recall of affectively toned stimuli: A meta-analytic review. Clin Psychol Rev. 1992;12:227–55. doi: 10.1016/0272-7358(92)90116-P

[52] Ecclestone A, Linden B, Boyes R, Stuart H. Comparing self-reported symptoms of anxiety and depression among Canadian post-secondary students to the general Canadian population during the COVID-19 pandemic: A national repeated cross-sectional trend analysis. Sage Open. 2025;15. doi: 10.1177/21582440251389262

