# Supplementary material for: Supporting recovery: Intersections of health, belonging, and help seeking after a campus mass casualty event
Source: PLoS One. 2026 Jul 29;21(7):e0353453. doi: 10.1371/journal.pone.0353453 (PMC13419175; doi:10.1371/journal.pone.0353453)
Supplement: Attachment — Submitted filename: Response to Reviewers.docx [file pone.0353453.s002.docx]

1. Please ensure that your manuscript meets PLOS ONE's style requirements, including those for file naming.

**Thank you for the comment. We have reviewed the PLOS ONE style requirements and modified all areas where formatting and naming conventions so they are now aligned with the journal.**

2. Please note that your Data Availability Statement is currently missing the a direct link to access each database. If your manuscript is accepted for publication, you will be asked to provide these details on a very short timeline. We therefore suggest that you provide this information now, though we will not hold up the peer review process if you are unable.

**Thank you for the comment. We have added a link to the open science framework repository that contains the data and code for this manuscript. The link is currently set to view only, but will be released publicly upon acceptance.**

**https://osf.io/5ad62/overview?view_only=d36c33d0ebf943aeaf7ea75e29d52432**

3. Please include your full ethics statement in the ‘Methods’ section of your manuscript file. In your statement, please include the full name of the IRB or ethics committee who approved or waived your study, as well as whether or not you obtained informed written or verbal consent. If consent was waived for your study, please include this information in your statement as well.

**Thank you for the comment, we have included the university ethics approval and consent process specific to this study.**

**This information was added in the Methods section. (Revised text below).**

**The study was exempted by the Office of Research Integrity – Social Behavioral Institutional Review Board of the University of Nevada, Las Vegas (protocol code #UNLV-2024-129 – February 20, 2024). The study was classified as Category 2(i) as the information obtained was recorded by the investigator in such a manner that the identity of the human subjects cannot readily be ascertained, directly or through identifiers linked to the subjects. Informed consent was provided at the start of the online questionnaire, along with a trigger warning to inform participants of the inclusion of questions related to the on-campus shooting, which occurred approximately 6 months prior to survey distribution. Upon acceptance of the electronic informed consent, the self-administered survey collected demographic information including status (employee or student), grade point average (GPA) for students only, age, race, ethnicity, sex, gender identity, sexual orientation, height, and weight.**

***Response to Reviewer Comments***

Reviewer #1: Overall, the manuscript is presented poorly, and the described methodology is below par in quality. I highlight some of the discrepancies below. This manuscript needs major changes before it could reach the publication stage.

**Thank you for the opportunity to clarify our methodology and revise this manuscript for publication. Much has been revised to improve clarity and thanks to reviewer recommendations, we believe the work is much improved.**

Reviewer 1; Comment 1: In the opening statement in the Introduction section (lines 46-47) I'd recommend insert the phrase "since the 1960s" for better clarity.

**Thank you for the comment. We clarity of message is important to us. The sentence was rewritten for better clarity.**

**Propose change to manuscript based on comment:**

***From the 1960’s to the end of 2023, thirteen college campuses in the United States were targets of mass shootings resulting in 102 deaths, as well as numerous physical, and emotional injuries[1].***

Reviewer 1; Comment 2: Expand the first occurrence of GPA on line 121.

**Thank you for catching this. We have expanded GPA at its first occurrence.**

Reviewer 1; Comment 3: On line 105 the authors stated that the screening questionnaires were administered "....before a mass shooting and several months after." Obviously, the authors would not have predicted a MCE. Hence the description of this research study as "pre/post" (see line 159) appears to be somewhat misleading. It appears the initial data gathering step of this research process "pre" was done for a different reason. I'd recommend the authors provide some clarity on why the initial step of the screening was conducted, it's aims and objectives.

Lines 116-118, the authors state: "...... along with a trigger warning to inform participants of the inclusion of 118 questions related to the on-campus shooting." I suspect this process pertains only to the second step of data gathering ("post"). I'd recommend the authors separate the two steps of data gathering and describe the purposes separately. On lines 115-116 the authors state: "The questionnaire was 116 distributed between April 12, and May 11, 2024." Which step of the research was distributed between these dates? When was the other step distributed? Also, please describe the date on which the tragic event had occurred. I suspect it took place near the student union on 12/6 (based on information from tables 1 and 2). If so, please state it in the text of the manuscript.

**REPONSE TO ADDRESS DISCREPANCY IN SURVEY TIMELINE (reference for all reviewers)**

**Thank you for making us aware of this major oversight, which was raised consistently by all reviewers. In response, and based on the importance of this feedback, we have made significant revisions throughout the manuscript to improve clarity, transparency, and alignment between the study purpose, methods, and interpretation of findings. These changes extend beyond a single section and were implemented across the abstract, introduction, methods, results, and discussion to ensure that the scope of the study and the rationale for included variables are clearly articulated. We believe these revisions substantially clarify the study methodology, strengthen the manuscript and address the reviewers’ concerns. We regret that the survey design and reporting time frames were not described with sufficient clarity in the original submission, and we appreciate the reviewers’ comments.**

**In short, the complete survey was distributed once, after the MCE had occurred. Within the survey the PHQ-9 and GAD-7 are delivered twice, with each instance having a unique prompt for participants to recall their feelings before the MCE and after the MCE. When we originally presented survey scores as “pre-event” that was incorrect. The survey was not distributed prior to the event. Rather we asked participants to recall their pre-event feelings for the first instance of the PHQ-9 and GAD-7 and then recall them again as they feel currently, after the event.**

**We have revised language throughout the manuscript to clearly indicate that this was a cross-sectional survey focused on recall of past feelings, rather than a longitudinal study that distributed the survey pre and post MCE. The survey was administered between April 12, and May 11, 2024 after the MCE which occurred on December 6, 2023. And again, participants were asked to recall their pre-MCE feelings first and then recall their current (post-MCE) feelings. Differences in these different recall types were the focus of our analysis.**

**Specifically, participants were first asked to recall their state of mind from approximately six months before the mass casualty event, and complete PHQ-9 & GAD-7. The participants then reported their feelings a second time with the prompt to report their current level of depression through the PHQ-9 and anxiety as evaluated by the GAD-7.**

**As each reviewer has correctly pointed out, there were instances in the manuscript where these two timeframes were referred to as ‘pre’ and ‘post,’ which erroneously led readers to think that participants completed the PHQ-9 and GAD-7 at two different times. To clarify the timeline of this study and it’s reporting in the results the timeframes have been renamed, Pre-MCE Recall and Recent Recall to better describe when the survey was taken and what the outcomes of the survey represent.**

Reviewer 1; Comment 4: Given the relatively large proportion of 36 out of 198 (see line 180), it would help the readers to have information about how statistically different respondents who did not fully complete the surveys compared to respondents who fully completed the surveys (see lines 180-181). Moreover, did all 162 respondents who "fully completed the survey" did so on all the administered questionnaire items?

In table 1: there is an "unknown" category under "Perceived discrimination mean". Please explain what the authors meant by "unknown" here.

**Thank you for the comment and catching the “unknown” designation for the PEDQ questionnaire as reported in Table 1. These five individuals are listed as unknown because they did not complete that portion of the survey. These individuals have been removed from the data set (as seen in Table) and all analyses have been updated to reflect that change. Their removal had no effect on the primary and secondary results of the study.**

**To address your initial comment regarding possible demographic or other survey responses differences between completers and non-completers of the survey, 36 of the 41 participants did not respond beyond recording their role at UNLV (student, student and employee, or employee only). Therefore, we performed an analysis looking at how role at UNLV was associated with completion or non-completion of the study and observed no relationship. Among the 5 participants who completed more than their role at UNLV, but not the entire survey, each only partially completed the PEDQ. These five participants were similar to the completers of the survey, which leaves us with limited information on what about this questionnaire may have caused their limited response.**

**Proposed change in manuscript in response to comment (lines 213-218)**

**“Logistic regression was used to examine any relationship between those who completed or did not fully complete the survey, with survey completeness (yes/no) as the outcome variable and role at the university as the predictor variable. Summary statistics of those who partially complete the survey were calculated and compared to the sample carried forward to describe potential bias in partial completion. “**

**“Completer analysis demonstrated that those who completed the survey versus those who did not complete the survey was not related to their role at the university (χ(2) = 3.94, p = 0.14). Five participants did complete a portion of the survey beyond reporting of their initial role. Among these partial completers, each reported similar demographic and survey responses like those who fully completed the survey. Interestingly, each did not fully complete the PEDQ, but we have no information on why this questionnaire within the survey was left incomplete among these participants.”**

Reviewer 1; Comment 5: On lines 220-221: the authors state: "Furthermore, there was also a treatment for anxiety and depression by time interaction.....". Please explain the nature and duration of this "treatment" in detail. Did all the survey respondents received the same "treatment" for exactly same duration of time? When did the treatment start and when did it end?

**The question, “Are you currently taking medication for or participating in counseling or psychotherapy for the treatment of depression or anxiety?” is further specified on lines 142–143 and was intended to assess individual participation in medication use or therapy. No intervention was performed as part of this study.**

**Updated text in the manuscript methods**

**“Participants were asked whether they were currently using antidepressant medication or engaged in counseling or psychotherapy for depression treatment.”**

Reviewer 1; Comment 6: On line 399 the authors themselves acknowledge that their study was "cross-sectional" in nature. However, throughout the manuscript the study design was highlighted as "pre/post" which essentially is longitudinal design. It would help the reviewers if the authors could clarify on aforementioned discrepancies.

**Thank you for the comment and attention to detail on this point. Please see our complete response to this discrepancy as you have brought in your earlier comment. The authors appreciate the reviewer’s feedback and regret the lack of clarity on this point in the original submission. This was a cross-sectional study intended to provide insight into factors that may support or hinder campus recovery following a mass shooting, an area in which the existing research remains limited.**

Reviewer #2: This is a robust and needed study, regardless of the relatively small response number. I found this interesting to read as it expanded my awareness of the impact of MCEs.
As you consider edits, there are initials which need definitions, and in my search did not always find the correct definition: eCRF, R, NAAN.

**Thank you for this comment. We have reviewed the manuscript to ensure that abbreviations are used correctly and have limited their use where possible.** **eCRF is for Estimated Cardiorespiratory Fitness, R is for the statistical programming language R, and NAAN is for Native Alaskan/American Indian.**

Reviewer 2; Comment 1: The use of figures and tables helps to support the findings. However, their number was a bit overwhelming. I found Figure 5 most meaningful. Please correct the labeling on figures 1, 2, 3, 4, and 6 for clarity. Table 1 requires review for punctuation corrections.

**Thank you for the comment and we appreciate the keen attention to detail in presentation of results. We have combined figures to now have 5 in total and have removed the model result tables to provide a cleaner and better reading experience. Rest assured that all model results can be viewed in the open science framework repository that the data and code that produced the results will be stored, if prospective readers are interested in the granular level outcomes of this research.**

Reviewer 2; Comment 1: Initially, I thought participants completed two surveys: one before the MCE and one after – I was left wondering how one knew when “before” an MCE would be. Rather, this was a single survey of participant reflections upon their experiences before and after the MCE. Perhaps it’s more accurate that respondents reflected on their experiences prior to and after the MCE and completed a self-assessment survey.

**Thank you for the comment and keen attention to detail in this regard. It is an area brought up by each reviewer. We have addressed this comment at length in an early comment by Reviewer 1, please see above, and we invite you to read that comment for the complete response. In short, data collection occurred between April 12 and May 11, and participants were asked to recall their state of mind prior to the shooting as well as during the two weeks preceding survey completion. We regret the lack of clarity in the original submission and have addressed this concern through substantial revisions throughout the manuscript. We have now shifted language throughout the manuscript to reflect that measures of PHQ-9 and GAD7 are of participant recall before and after the MCE, and not that these surveys were delivered before and after the MCE.**

Reviewer 2; Comment 2: Comparing facets of belonging, race, anxiety, depression, and race on a college campus to what supports individuals seek is important information regardless of experiencing an MCE. And yet crucial to knowing as universities prepare for such situations. Thank you for undertaking this important work.

**We would like to express our deep gratitude for this comment. It is our hope that better understand of campus resilience and areas of need after such unfortunate events can help in response and recovery for future events.**

Reviewer #3: This manuscript examines how students and staff at a minority-serving institution (MSI) were affected by a mass shooting event (MSE), with attention to changes in mental health, sense of belonging, and fitness. The manuscript also examined whether and how study participants sought help after the MSE. The topic is timely and important. There remains a notable gap in survey-based research that systematically examines the range of social, emotional, and psychological sequelae following MSEs. The authors’ focus on both students and staff is also potentially valuable, as staff experiences are often underexamined in this literature.

However, despite the importance of the topic, the manuscript currently has a number of conceptual and methodological weaknesses that limit its interpretability and contribution. Most critically, there is significant ambiguity surrounding the study design, timing of data collection relative to the MSE, and whether the data are longitudinal or cross-sectional. These issues, along with concerns about construct selection, analytic organization, and measurement reporting, must be addressed before the manuscript can make a meaningful contribution to the field. More details are provided below.

**Thank you for the comment and meaningful appraisal of this manuscript. We agree with each point introduced in this broader comment and below we address each point-by-point.**

Major Concerns

Reviewer 3; Comment 1: The manuscript does not clearly specify whether participants were surveyed at two time points (pre- and post-MSE) or whether different participants were surveyed at different times. At several points, the language implies a pre–post design with the same participants, yet the limitations section describes the study as cross-sectional. This inconsistency creates substantial confusion and makes it difficult to interpret any claims about “change.”
If participants were not surveyed at two time points, this must be stated explicitly and consistently throughout the manuscript. A cross-sectional study, by definition, implies that participants were surveyed only once. Any language suggesting within-person change (e.g., pre–post effects) should be removed or revised accordingly. All language that implies causality, such as “impacted” should be removed. For instance, replace “impacts” with “is associated with”.

Critically, the timing of survey administration relative to the MSE must be made clear. Information about when baseline data were collected, when the MSE occurred, and when post-event data were collected should be summarized in the paragraph immediately preceding the Methods section and then described in detail within the Methods. The details can be done without specifying the date of the MSE (e.g., report “average days elapsed between MSE and post-test date” and use as a control) should the authors want to blind the date of the MSE so that the institution can remain anonymous. Without this information, readers cannot assess the internal validity of the study or the plausibility of temporal interpretations.

**Thank you for the comment and keen attention to detail in this regard. It is an area brought up by each reviewer. We have addressed this comment at length in an early comment by Reviewer 1 and we invite you to read that comment for the complete response. In short, data collection occurred between April 12 and May 11, and participants were asked to recall their state of mind prior to the shooting as well as during the two weeks preceding survey completion. We regret the lack of clarity in the original submission and have addressed this concern through substantial revisions throughout the manuscript. We have now shifted language throughout the manuscript to reflect that measures of PHQ-9 and GAD7 are of participant recall before and after the MCE, and not that these surveys were delivered before and after the MCE.**

Reviewer 3; Comment 2: Relatedly, the statistical approach should reflect the actual design. If the data are cross-sectional, the analyses must be framed accordingly, and the manuscript should include a clear discussion of sample size, group comparability, and the limitations of between-group comparisons over time.

**Thank you for the comment. We have modified the statistical approach to better reflect the study design. We believe much of the ambiguity around the analysis has been resolved through better contextualization of when the survey was administered. Specifically, this was a within subject design to look at the differences in perceived anxiety and depression as recalled by participants. We have also included a paragraph that addresses sample size.**

**Additional text in statistical analysis section for sample size justification**

**“**Given the nature of this research, there was not a priori power analysis to inform the appropriate sample size given it’s spontaneous and challenging conditions. Additionally, data collection was considered to be time sensitive, i.e. survey responses need to be collected over a consolidated amount of time to prevent any recall bias. However, using typical effect size standards [40] the aim was to be adequately powered to detect moderate effect sizes (r = .3) which require a total sample size of 82.”

Reviewer 3; Comment 3: A second major concern is that the manuscript reads as though this study may have been embedded within a larger research project that was already underway when the MSE occurred. If this is the case, it is essential to clarify why participants were being surveyed in the first place, what they were told about the study, and whether the MSE-related questions were added after the event.

**We understand why the original draft may have incorrectly inferred this conclusion. Indeed, the study included only one data collection point, specifically designed to examine the responses of students and employees to this event and to explore various constructs that may have influenced its impact.**

Reviewer 3; Comment 4: Details about the broader study context—including recruitment procedures, study aims at the time of consent, and whether the MSE analysis was planned or opportunistic—should be explicitly described. This information is necessary for evaluating potential selection bias, response bias, and the interpretability of findings.

**Thank you for these valuable suggestions. We have revised the purpose statement and included information about the survey distribution.**

**Text in materials and methods in reference to survey distribution**

**“The survey was administered between April 12 and May 11, 2024. Within the survey, the PHQ-9 [35] and GAD-7 [36] were each administered twice. Participants first completed both instruments by recalling their mental health status during the period prior to the campus shooting. They then completed the PHQ-9 and GAD-7 a second time with reference to the two weeks immediately preceding survey completion, which was used to assess current mental health status.”**

Reviewer 3; Comment 5: A third major concern is that the set of constructs examined in the manuscript lacks conceptual cohesion. While changes in mental health outcomes such as depression and anxiety are clearly relevant following an MSE, the inclusion of fitness is insufficiently justified.

**More information has been included in the revisions to justify the inclusion of cardiorespiratory fitness.**

Review 3; Comment 6; The manuscript does not make a compelling theoretical or empirical case for why fitness should be examined as a primary outcome alongside mental health and belonging. The paper would be substantially strengthened by focusing on psychological functioning (e.g., depression, anxiety, distress) as the primary outcomes, with constructs such as belonging and fitness examined as moderators or mediators. This approach would improve conceptual clarity and align the analyses more closely with existing trauma and stress literature.

**We appreciate this valuable suggestion and carefully considered the recommendation. At this time, we respectfully request retaining estimated cardiorespiratory fitness as a variable that may moderate symptoms of depression and anxiety following the mass shooting. Prior research consistently demonstrates that higher levels of fitness are associated with lower levels of depression and anxiety, and a lack of fitness is correlated with increased levels of the same. Long-term behavioral patterns related to physical activity and sedentary behavior have a meaningful influence on mental health outcomes in many contexts. Given the exploratory nature of this study, inclusion of cardiorespiratory fitness may offer important insight for university stakeholders considering strategies to address trauma on campus. Additionally, most universities already invest substantial resources in fitness and recreation infrastructure, and findings related to treatment preferences suggest that students are receptive to fitness-based and movement-oriented interventions as potential forms of support.**

Reviewer 3; Comment 7: Similarly, the inclusion of the research question related to help-seeking behavior after the MSE appears underdeveloped and somewhat tangential. This research question is not well integrated into the manuscript’s theoretical framework and reads as an afterthought—and is also too much material/too many topics to cover with the care they need. The authors may wish to consider focusing this paper on psychological and social outcomes following the MSE and reserving help-seeking analyses for a separate manuscript.

**Thank you for this thoughtful suggestion. While we understand the reviewer’s concern and acknowledge that preferences for counseling or alternative approaches such as meditation or exercise are not the primary outcomes of the present study, we respectfully believe this information should remain in the manuscript. The central aim of this work is not only to document changes in depression and anxiety following a mass casualty event, but also to offer data that may inform practical, campus-level responses to such a tragedy. Understanding students’ stated preferences for potential support options provides applied context for how universities might engage and reach their communities during recovery, particularly when traditional counseling services may be strained or underutilized. Although these preference items are not directly linked to the measurement of depression and anxiety severity, they offer actionable insight for stakeholders seeking to implement supportive, trauma-informed resources that align with student interests and existing campus infrastructure. For these reasons, and given the exploratory and translational nature of the study, the authors believe retaining this information strengthens the relevance and utility of the findings for campus communities navigating similar events.**

Reviewer 3; Comment 8: Fourth, while the manuscript provides solid information regarding the reliability and validity of the belonging measure, similar information is missing or insufficient for other key measures. For all scales used, the authors should report as much information about reliability and briefly describe evidence of prior validity and note any potential areas where measures are new or not yet fully validated and validated with a minority sample. Given the centrality of measurement quality to the manuscript’s claims, this information is essential.

**Based on this important comment, we have made serious revisions to the article and have included the requested information in the Methods section. The information is outlined below.**

**“Depressive symptom severity was assessed using the PHQ-9, a brief, validated self-report measure widely used for screening, monitoring, and quantifying depression severity in both research and clinical settings. The PHQ-9 consists of nine items reflecting Diagnostic and Statistical Manual of Mental Disorders, Fourth Edition (DSM-IV) criteria for major depressive disorder and assesses symptom frequency over the past two weeks. Items are scored from 0 to 3 (not at all, several days, more than half the days, or nearly every day), yielding a total score ranging from 0 to 27. Standard cut points categorize scores as follows: 0–4 (no depression), 5–9 (minimal symptoms), 10–14 (mild depression), 15–19 (moderately severe depression), and ≥20 (severe depression). A PHQ-9 score of 10 or higher demonstrates strong diagnostic accuracy for major depression, with both sensitivity and specificity of 88% [35].**

**Anxiety symptom severity was assessed using the GAD-7, a seven-question validated self-report measure widely used to screen for anxiety severity[36]. The GAD-7 consists of seven items reflecting core symptoms of generalized anxiety disorder and assesses symptom frequency over the past two weeks. Items are scored from 0 to 3 (not at all, several days, more than half the days, or nearly every day), yielding a total score ranging from 0 to 21. Standard cut points categorize scores as follows: 0–4 (minimal anxiety), 5–9 (mild anxiety), 10–14 (moderate anxiety), and ≥15 (severe anxiety). A GAD-7 score of 10 or higher has demonstrated good diagnostic accuracy for generalized anxiety disorder, with a sensitivity of 89% and specificity of 82% [36].**

**Perceived discrimination was assessed using the Brief Perceived Ethnic Discrimination Questionnaire–Community Version (Brief PEDQ-CV), a 17-item abbreviated form of the original 70-item PEDQ-CV [32]. Items assess the frequency of discriminatory experiences and are rated on a 5-point Likert scale ranging from 1 (never) to 5 (very often), with higher scores indicating greater perceived discrimination. A total PEDQ-CV score was computed by averaging responses across all 17 items. The Brief PEDQ-CV has demonstrated acceptable to strong internal consistency, with reported Cronbach’s alpha coefficients ranging from 0.65 to 0.88 in community and student samples[32]. Consistent with the Sense of Belonging Scale, the PEDQ-CV captured participants’ overall perceptions of discrimination at the time of survey completion.**

**Cardiorespiratory fitness was estimated using a validated and reliable survey instrument to examine responses to the trauma of the mass shooting event in relation to fitness level. Cardiorespiratory fitness reflects the cumulative effect of sedentary and physical activity behaviors. The eCRF measure developed by Nes et al. has demonstrated strong validity and has been shown to be comparable in accuracy to many field-based assessments of peak oxygen consumption (VO₂peak) [33,34]. The model was derived from cross-validation VO₂peak data from 4,367 healthy adults and has demonstrated acceptable accuracy for estimating sex-specific VO₂peak in outpatient and population-based settings[33,34]. The regression model includes age, body mass index (BMI), resting heart rate (RHR), and a physical activity index (PA-I). The PA-I was calculated from self-reported exercise frequency, duration, and intensity using a previously validated scoring system [33,34,39] and has shown moderate correlations with measured VO₂peak (r = 0.44 for men; r = 0.38 for women). Although the original model incorporated waist circumference, a BMI-based algorithm has been shown to produce comparable estimates (Nes et al., 2014). Given inconsistencies in self-reported waist circumference, the BMI-based model was used in the present study.**

**Sex-specific equations were applied as follows:**

**Men (R² = 0.59, SEE = 5.8): 92.05 − (0.327 × age) − (0.933 × BMI) − (0.167 × RHR) + (0.257 × PA-I)
Women (R² = 0.57, SEE = 5.1): 70.77 − (0.244 × age) − (0.749 × BMI) − (0.107 × RHR) + (0.213 × PA-I)**

**For each participant, estimated CRF (eCRF) was compared with age-predicted normative CRF values for healthy adults[33,40]. To enhance methodological rigor, eCRF estimates and normative values were cross-verified using the web-based calculator developed by the original authors. Both eCRF and the difference between individual eCRF and normative CRF values (DIFF) were included in subsequent analyses**.”

Less Major Concerns

Reviewer 3; Comment 9: The sample includes both students and staff, who are likely to differ substantially in exposure, role-related stressors, access to resources, and help-seeking norms. This reviewer suggests that student versus staff status should be examined explicitly as a moderator. Doing so would allow for more nuanced interpretation and would strengthen the manuscript’s contribution by carefully identifying and discussing whether the effects of the MSE differ across institutional roles. This is also another reason why an examination of help-seeking should not be included in this manuscript, as the help-seeking paths (and potential pathways) and reasons will greatly differ across students versus adult employees who are not students.

**Thank you for your comment and we agree that university role as a moderator is an important variable to investigate in terms of differential impacts to anxiety and depression in response to an MCE.**

Reviewer 3; Comment 10; A topic that needs more clarity is related to the IRB review. The manuscript notes that the study was considered IRB-exempt, but provides no explanation for this determination. Given that the study involves human subjects and aims to produce generalizable knowledge, further clarification is needed. The authors should explain the basis for the exemption (e.g., anonymous data collection, minimal risk, use of existing data). Providing this information would increase transparency and reassure readers that ethical considerations were appropriately addressed—particularly important given the sensitive nature of the topic (even with anonymity promised).

**Thank you for this suggestion and the time you took to review our work. We have added more specific information regarding the ethics review for this study and how informed consent was obtained.**

**“The study was exempted by the Office of Research Integrity – Social Behavioral Institutional Review Board of the University of Nevada, Las Vegas (protocol code #UNLV-2024-129 – February 20, 2024). The study was classified as Category 2(i) as the information obtained was recorded by the investigator in such a manner that the identity of the human subjects cannot readily be ascertained, directly or through identifiers linked to the subjects. Informed consent was provided at the start of the online questionnaire, along with a trigger warning to inform participants of the inclusion of questions related to the on-campus shooting, which occurred approximately 6 months prior to survey distribution. Upon acceptance of the electronic informed consent, the self-administered survey collected demographic information including status (employee or student), grade point average (GPA) for students only, age, race, ethnicity, sex, gender identity, sexual orientation, height, and weight.”**

**We include university role (employee only versus non-employee only) as moderator in the GAD7 and PHQ-9 results and did not observe a statistically significant result. Inclusion of this analysis and result are included in the manuscript. However, due to the relative imbalance of employee to student within the sample, we also completed a sub-group analysis among employees only, albeit with much simpler models as to avoid overfitting. Results from this sub-group analysis has also been included in the paper as well.**

Reviewer 3; Comment 11: Last, the results section is difficult to follow, as findings related to different constructs are interwoven without a clear analytic structure. A more coherent approach would be to first present results related to changes in mental health outcomes, followed by analyses examining moderators or mediators (e.g., belonging, fitness, staff vs. student status). Reorganizing the results section in this way would greatly enhance clarity and help readers understand what was found and for whom.

**Thank you for the comment as we strive to present these results as clearly as possible. We have consolidated figures so that each figure is representative of a distinction section. Also, we have removed the large and cumbersome results tables that were difficult to interpret as stand-alone tables. However, all detailed results can be found in the online repository where the data and code that produced the results will be hosted. We agree with your approach and have re-structured the results to follow the presentation you have recommended, by first presenting aggregate differences, then observed moderators, and finally attitudes of available services.**

To ensure your figures meet our technical requirements, please review our figure guidelines: <https://journals.plos.org/plosone/s/figures>

You may also use PLOS’s free figure tool, NAAS, to help you prepare publication quality figures: <https://journals.plos.org/plosone/s/figures#loc-tools-for-figure-preparation>.

NAAS will assess whether your figures meet our technical requirements by comparing each figure against our figure specifications.
