# Supplementary material for: Supporting recovery: Intersections of health, belonging, and help seeking after a campus mass casualty event
Source: PLoS One. 2026 Jul 29;21(7):e0353453. doi: 10.1371/journal.pone.0353453 (PMC13419175; doi:10.1371/journal.pone.0353453)
Supplement: Attachment — Submitted filename: PLOS notes 5 1 26 Final.docx [file pone.0353453.s003.docx]

6. Review Comments to the Author

Please use the space provided to explain your answers to the questions above. You may also include additional comments for the author, including concerns about dual publication, research ethics, or publication ethics. (Please upload your review as an attachment if it exceeds 20,000 characters)

Reviewer #2: I appreciate your thorough response to 3 reviewers’ comments and the attention to revising the manuscript to increase readability and transparency. In this second reading of the manuscript, I am reminded that there are multiple authors, and each one has a voice. Below in my later comments I identify clear language used which might inform your responses to preceding comments. Thank you for considering this feedback:

Line 36-37 In the abstract, “Increases in depression were linked to lower pre-MCE anxiety, higher PEDQ-CV scores, and receiving current treatment for mental health concerns.” So those who had low anxiety prior to the MCE, overall higher perceived discrimination AND receiving current treatment had an increase in depression post-MCE? **Please confirm how are these three things are related to an increase in depression.**

**Thank you for the comment and sincere feedback! We have modified the reporting of the results in the abstract to be more clear on the direction of each variable on our measured outcome of depression. Below is the revised text in the abstract to clarify the link.**

*Increases in depression (PHQ-9 scores) from pre-MCE to recent recall were larger among respondents with lower pre-MCE anxiety (GAD-7 scores), higher perceived discrimination (PEDQ), and those currently receiving treatment for anxiety or depression; respondents not receiving treatment showed no significant change.*

*Lines 71: Editorial suggestion to cut “there are” for increased readability: “The trauma of exposure to an active shooter and loss of life in one’s normal living environment may exacerbate these issues,[2]and other factors may help or hurt the recovery process.”*

*Line 72: Editorial suggestion to cut “also” for increased readability: “Low fitness is associated with poorer mental health outcomes[14]…”*

**Thank you for the suggestion! We have updated the text accordingly.**

*Line 110: In the prior line, you mention retrospectively reporting symptoms, but in 110 do not specific the length of the period prior to the mass shooting. “…from the period prior…” tho you go on to specify “during the two weeks immediately preceding survey completion” in line 111. If you’re going to specify one time period, please specify the other.*

**Thank you for the comment! We have specified the recall time frame, which was (1) the two weeks immediately preceding the mass casualty event (approximately 4–5 months prior to survey administration), and (2) the two weeks immediately preceding completion of the questionnaire.**

*Line 112: You identify “survey instruments” but because this is not pre/post, it’s ONE long survey incorporating multiple measurements, yes? Asking folks to reflect twice on their symptoms, before the MCE and after. Please clarify that you have incorporated multiple questionnaires into one self-report survey. (Line 263 uses “questionnaire within the survey” which could provide clarity in line 112).*

**Thank you for this insight, indeed this was not clear.** **We have revised the text throughout the manuscript to detail the nature by which and when each measurement was collected.**

*Line 121: Appreciate the additional information in this section, however not every reader will understand what a “Category 2(i)” is. At my university, we would call the description that followed as “Exempt.” Perhaps adding that term in parentheses would be a sufficient explanation.*

**Thank you for this comment! We have updated the manuscript accordingly.** **Please see below for updated text.**

The study was classified as exempt (Category 2(i) under 45 CFR 46.104(d)(2)) as the information obtained was recorded by the investigator in such a manner that the identity of the human subjects cannot readily be ascertained, directly or through identifiers linked to the subjects.).

*Lines 136-141: Although an improved survey description, it still sounds like these measurements/instruments are being administered separately, rather than the self-administered survey, it two parts, included/repeated such measurements : for the X time period retrospective, and then the more immediate reflection of after the MCE. Or somesuch. This section needs additional clarity. Note: subsequently, you describe the PHQ-9, GAD-7, SBS (line 164), PEDQ (line 176), CRF (line 185), and PA-I (line 193), however you do not include the last 4 items in lines 136-141. If you are using this section to summarize the survey, please include all instruments/questionaires incorporated into the single survey for clarity*.

**This was an unfortunate oversight and your comments have prompted us to update the language, which will clarify the multiple components of the singular questionnaire. The entire passage has been updated. Please see below for updated text.**

Upon providing electronic informed consent, participants completed a multi-faceted, self-administered questionnaire that included several validated instruments, including the Patient Health Questionnaire-9 (PHQ-9)[34] and the Generalized Anxiety Disorder-7 (GAD-7)[35]. Notably, the PHQ-9 and GAD-7 were the only measures requiring retrospective recall. For these instruments, participants reported their mood during two time periods: (1) the two weeks immediately preceding the mass casualty event (approximately 4–5 months prior to survey administration), and (2) the two weeks immediately preceding completion of the questionnaire. All other measures included in the questionnaire, including the Student Belongingness Survey (SBS)[36] and the Perceived Ethnic Discrimination Questionnaire (PEDQ)[32], assessed participants’ current state only.

Participants were also asked for demographic information, including university status (employee or student), grade point average (GPA; students only), age, race, ethnicity, sex, gender identity, sexual orientation, height, and weight. Body mass index (BMI) was calculated from self-reported height and weight using the formula weight (kg) / [height (m)]². A Physical Activity Index (PA-I) and estimated cardiorespiratory fitness (eCRF) were assessed using validated algorithms[33,37]. Participants reported whether they were currently using antidepressant medication or engaged in counseling or psychotherapy for depression treatment. Finally, participants stated their willingness to attend counseling sessions at the university (yes/no) and their interest in learning about alternative approaches, such as meditation and exercise (yes/no).


*Line 185: You lost me. Is the CRF estimation part of your analysis or self-reported data collection? It looks like the PA-1 and POSSIBLY the RHR was collected as part of the self-report survey. Please clarify what was collected in the survey prior to diving into how the information was analyzed… Please clarify if you used self-report information to generate the eCRF.*

**Thank you for your comment and the opportunity to clarify! The algorithm used to calculate eCRF incorporates the PA-I and is described in greater detail in the Methods section. To improve clarity, we have revised the first mention in the Methods. The updated text is provided below:**

A Physical Activity Index (PA-I) and estimated cardiorespiratory fitness (eCRF) were derived from participant data reported in the survey which are entered into validated algorithms for calculations (see further details below) [33,37].

...Cardiorespiratory fitness was estimated using a validated and reliable algorithm to examine responses to the trauma of the mass shooting event in relation to fitness level. Cardiorespiratory fitness reflects the cumulative effect of sedentary and physical activity behaviors. The eCRF measure developed by Nes et al. has demonstrated strong validity and has been shown to be comparable in accuracy to many field-based assessments of peak oxygen consumption (VO₂peak) [33,37]. The model was derived from cross-validation VO₂peak data from 4,367 healthy adults and has demonstrated acceptable accuracy for estimating sex-specific VO₂peak in outpatient and population-based settings[33,37]. The regression model includes age, body mass index (BMI), resting heart rate (RHR), and a physical activity index (PA-I). The PA-I was calculated from self-reported exercise frequency, duration, and intensity using a previously validated scoring system [33,37,39] and has shown moderate correlations with measured VO₂peak (r = 0.44 for men; r = 0.38 for women). Although the original model incorporated waist circumference, a BMI-based algorithm has been shown to produce comparable estimates [37]. Given inconsistencies in self-reported waist circumference, the BMI-based model was used in the present study.

Sex-specific equations were applied as follows:

Men (R² = 0.59, SEE = 5.8): 92.05 − (0.327 × age) − (0.933 × BMI) − (0.167 × RHR) + (0.257 × PA-I)
Women (R² = 0.57, SEE = 5.1): 70.77 − (0.244 × age) − (0.749 × BMI) − (0.107 × RHR) + (0.213 × PA-I)

For each participant, estimated CRF (eCRF) was compared with age-predicted normative CRF values for healthy adults[33,40]. To enhance methodological rigor, eCRF estimates and normative values were cross-verified using the web-based calculator developed by the original authors. Both eCRF and the difference between individual eCRF and normative CRF values (DIFF) were included in subsequent analyses.

*Lines 208-209: “subsequent analyses” re: eCRF are references here but I don’t see them in the statistical analyses and minimally in the results section (Lines 343, 385, solely related to employees).*

**Thank you for this important observation! The reviewer is correct that, in the prior version, eCRF and the eCRF–normative difference (DIFF) were introduced in the Methods as variables to be carried into “subsequent analyses,” but the corresponding results were not consistently reported. We have addressed this in two ways. First, we standardized terminology so that eCRF and DIFF are referred to consistently throughout the Methods, Statistical Analysis, and Results, including explicit listing of cardiorespiratory fitness in the all-respondent, student sub-group, and employee sub-group analyses. Second, we added explicit reporting of cardiorespiratory fitness in the all-respondent PHQ-9, all-respondent GAD-7, student sub-group, and employee sub-group results paragraphs, so that readers can see these variables were tested even when associations were not statistically significant.**

Line 229: Reference to “(pre/post)” to be removed and clarified to avoid reader confusion. At one point, 6 months of recall vs. 2 weeks immediately prior to the survey is mentioned earlier in the manuscript. For consistency, repeat those time frames throughout the study to remind the reader to the time frames participants were to recall.

**Thank you for the comment! We have updated the materials and methods section to be explicit with how we label the different types of recall that were measured during the survey. We carry this labeling forward across the statistical analysis and results section for consistency. Revised text is displayed below.**

Upon providing electronic informed consent, participants completed a multi-faceted, self-administered questionnaire that included several validated instruments, including the Patient Health Questionnaire-9 (PHQ-9)[34] and the Generalized Anxiety Disorder-7 (GAD-7)[35]. Notably, the PHQ-9 and GAD-7 were the only measures requiring retrospective recall. For these instruments, participants reported their mood during two time periods: (1) the two weeks immediately preceding the mass casualty event (pre-MCE recall, approximately 4–5 months prior to survey administration), and (2) the two weeks immediately preceding completion of the questionnaire (recent recall).

Lines 227-234: This is a long section that begins with identifying independent variable, covariates, and ends with “were used as control variables.” Is there a period missing somewhere? Please clarify that which were control variables and that which were not.

**Thank you for the comment! We have revised this section for clarity of what was the primary variables of interest and covariates. Revised section below.**

*A linear mixed effects model with random intercepts for participant was used to examine individual differences in PHQ-9 with primary independent variables of recall (pre-MCE recall/current recall), pre-MCE recall GAD-7 scores, current treatment status for anxiety or depression (yes/no), difference in actual (eCRF) to perceived (Hunt Norm) fitness, log-transformed mean PEDQ, and role (student, student and employee, and employee only). The model also included control variables of participant age, Sex (Male, Female), Sex/Gender Minority (non-SGM versus SGM), race, Latino/Hispanic ethnicity, and location at the time of the event (on-campus, near campus, off-campus).*

Line 235 is the clearest explanation of how the survey was presented “between recall (pre-MCE verses current)” – consider using this explainer throughout.

**Thank you for the comment and emphasizing that use for consistency. We have now updated the manuscript to maintain that explanation of what the survey measured throughout.**

Line 263: Again, the clearest explanation of a measurement within the survey – consider using throughout: “questionnaire within the survey”

**Thank you! We have now updated the manuscript to make clear this explanation.**

Line 273: Table 1 Participant Demographics. You have included measurement responses along with participant demographics. And even then, you do not include ALL of the measurement response. Perhaps separate the information between simple demographics and questionnaire response means.

**Thank you for the comment! We have clarified the table into separate sections for demographics versus responses and also included additional measurement responses which were previously not included.**

Line 276: BRAVA! “…pre MCE and recent recall” is the best descriptor I’ve read yet! See if this language can be incorporated throughout for increased clarity.

**Thank you! We have now incorporated this language throughout.**

Line 285: Yes! “Recall difference” makes your research so much clearer. Thank you!

**Thank you! This helps us confirm we are on the right track.**

Line 301-302: Increased clarity needed. Perhaps just restating the previous sentence about the relationship between pre-MCE recall GAD-7 and both recall time points for PHQ-9. (Or perhaps I will better understand the results section when I get to the discussion section.) ;)

**Thank you! We agree this can be made clearer. Below is the revised text.**

The interaction between recall period and pre-MCE GAD-7 score was significant (β = -1.09, 95% CI = [-1.83, -0.35], p = .006, Figure 2A): participants with lower pre-MCE GAD-7 scores showed the largest increases in recent-recall PHQ-9, while participants with higher pre-MCE GAD-7 scores showed little change.

Line 309: Typo? “Furthermore there was also a treatment…” Instead of treatment, interaction?

**That is correct! We have updated for clarity.**

Furthermore, there was also an anxiety and depression by recall interaction

Line 315: Replace “pre/post”

**Thank you for catching this! We have updated it to our current terminology for consistency and clarity. Pre-MCE recall/recent recall.**

Line 392-393: This feels important to restate “…employees who reported higher perceived discrimination also had higher [anxiety]…”

**Thank you! We have revised to make clear what the measure represents for better interpretation.**

Lines 440-446: This feels like a significant finding. Consider adding the information from lines 392-393.

**Thank you for the comment! We have reiterated the finding within the discussion to better elaborate the results of each analysis. Revises text can be seen below.**

However, the magnitude of the difference was dependent on the level of anxiety and depression experienced before the shooting, perceived discrimination, and participation in treatment for anxiety and/or depression. Specifically, among students perceived sense of school belongingness also modified individual depression, while among employees perceived discrimination was significantly to their anxiety.

Lines 463-471: discuss the importance of physical activity on mental health but lacks specific information from respondents. There is no discussion about eCRF, BMI, or the PA-I information gleaned from participants. Regardless if cardiovascular fitness is important to participants, please include this in the discussion. Fitness levels of participants were not part of the discussion.

**We appreciate this comment! Although estimated cardiorespiratory fitness was not significantly associated with the outcomes in our analyses, we included this variable based on its well-established relevance to overall health and student well-being. In the Discussion, we have clarified our rationale for including this measure and noted that the lack of significant findings in our sample does not necessarily diminish its potential importance, but may reflect sample characteristics, measurement limitations, or other unmeasured factors. In addition, we reorganized the Discussion to first focus on students’ preferences for seeking counseling or alternative interventions, such as exercise, to improve the overall flow and cohesion of the section. We have added text to better explain these points.**

Although the results of this study did not include significant associations between fitness and other variables, a substantial body of evidence indicates that improved cardiorespiratory fitness, a result of regular physical activity, improves mental health [44-46]. Previous research suggests that low fitness is linked to increased depressive symptoms in college students[19], and exercise is shown to be a crucial factor in maintaining their mental and cognitive well-being [47-49].

This manuscript is getting closer to publication. I recommend addressing these revisions prior to resubmitting. This is an important study! Thank you for the opportunity to read your revised manuscript.

**Thank you for your thoughtful and detailed review. We hope the revisions made here improve the clarity and impact of this work.**

Reviewer #3: Although the authors acknowledged the cross sectional design, a central remaining concern involves the use of retrospective self-reports to approximate pre-event functioning within what is now clarified to be a cross-sectional design. While the authors have improved clarity around the study design, the analytic approach still relies on participants’ recall of their pre-MSE mental health, belonging, and related states to draw inferences about change. This approach raises significant concerns about measurement validity.

A large body of methodological and psychological research has demonstrated that retrospective reports of prior states—especially following traumatic or highly salient events such as a mass shooting—are systematically biased (see for instance review in Faul & LaBar, 2023). Recall bias, mood-congruent memory, and cognitive reconstruction processes can lead individuals to reinterpret their past functioning in light of their current experiences. Basically, people recall past experiences in ways that are consistent with their current mood. For example, individuals experiencing distress post-event may recall their pre-event functioning as better than it actually was, thereby inflating perceived declines; alternatively, some may normalize past distress, attenuating perceived change. These processes are well-documented and pose a direct threat to the validity of inferred pre–post differences when based on retrospective recall. Furthermore, for people have experienced depression or who are currently depressed, recall may be even less reliable and valid measures. See for instance:Brand et al., 1992 in Journal of Affective Disorders.

Accordingly, the “pre” measures in this study should not be treated as equivalent to true baseline data, and the resulting comparisons cannot be interpreted as estimates of within-person change. At most, they capture perceived or reconstructed change, which is conceptually distinct. This reviewer notes that the authors do utilize the term "perceived" change in this revision, but nonetheless, it remains very difficult to interpret what the change signifies and hence the implications of these finings. **Basically, this distinction has important implications for how the findings are framed and interpreted. The manuscript would be strengthened by explicitly acknowledging these limitations in the relevant places (methods, limitations and conclusion).**

Faul, L., & LaBar, K. S. (2023). Mood-congruent memory revisited. Psychological review, 130(6), 1421–1456. <https://doi.org/10.1037/rev0000394>

**Thank you for this insightful and valuable comment. We greatly appreciate the suggested reference, which enhanced our understanding of mood-congruent memory and its relevance to our study design. In response, we have incorporated this concept into the manuscript and included additional supporting literature. We also expanded this section to note that pre-event recall scores in our sample were comparable to those reported in prior studies of university students, which provides some support for the validity of our retrospective assessments and suggests that substantial mood-related inflation may be less likely. This addition has strengthened the discussion of potential recall bias and improved the overall rigor of the paper.**

**Discussion**

Another consideration is mood-congruent memory, where prior research suggests that individuals may preferentially recall information consistent with their current emotional state, which could influence how participants reconstruct past experiences[50,51]. This may have affected participants’ pre-MCE recall, particularly among those reporting higher anxiety or depression at survey completion. However, prior research examining anxiety and depression among university students suggests that pre-MCE recall scores observed in our sample are comparable to those reported in the literature[52]. This comparability supports that the recall differences observed here are valid and suggests that participants' retrospective reports were less influenced by mood-based bias than expected. Specifically, if participants were systematically inflating the positivity of their pre-MCE experiences, we would expect lower levels of anxiety and depression than typically reported, which we did not observe. Conversely, if respondents were normalizing or minimizing distress from the MCE, we would expect a smaller difference between pre-MCE and recent recall reports. Together, these patterns suggest that mood-congruent memory may not substantially explain the recall differences observed here, though this interpretation warrants caution.

**Limitations**

Fifth, mood-congruent memory theory suggests that recall is not neutral, but rather biased toward information that is consistent with an individual’s current emotional state[50,51]. Accordingly, participants with elevated depressive or anxiety symptoms at the time of assessment may have preferentially recalled mood-congruent experiences from the pre-event period, potentially inflating self-reported depression and anxiety. The use of validated instruments (PHQ-9, GAD-7) may help mitigate, but not eliminate, this limitation. As well, the comparability of pre-event recall scores in our sample to those reported in prior studies of university students suggests that substantial mood-related inflation of retrospective symptoms may be less likely[52].

**Conclusion**

Overall, these findings highlight the complex interplay between trauma, mental health, and campus climate. **These findings are also subject to recall bias, particularly mood's influence on retrospective reporting of pre-MCE symptoms.** Beyond offering opportunities for mental health counseling, institutional efforts to promote belongingness, address discrimination, and expand mental health resources are critical in mitigating the psychological consequences of a MCE. Future research could explore longitudinal outcomes to determine the long-term effectiveness of different interventions and assess how various student subgroups may require tailored support strategies. By adopting a holistic approach to student well-being, universities can better equip their community to cope with trauma and foster a resilient campus community.
