# Supplementary material for: Supporting recovery: Intersections of health, belonging, and help seeking after a campus mass casualty event
Source: PLoS One. 2026 Jul 29;21(7):e0353453. doi: 10.1371/journal.pone.0353453 (PMC13419175; doi:10.1371/journal.pone.0353453)
Supplement: Peer review history

# Peer review: Decision Letter 0

**DOI:** 10.1371/journal.pone.0353453.r001

**Authors:** Janet Rosenbaum

2 Jan 2026

PONE-D-25-33879

Supporting recovery: Intersections of health, belonging, and help seeking after a campus mass casualty event

PLOS One

Dear Dr. Jalene,

Thank you for submitting your manuscript to PLOS ONE. After careful consideration, we feel that it has merit but does not fully meet PLOS ONE’s publication criteria as it currently stands. Therefore, we invite you to submit a revised version of the manuscript that addresses the points raised during the review process.

Please address all constructive comments from the reviewers as you prepare your revision.

Please submit your revised manuscript by Feb 16 2026 11:59PM. If you will need more time than this to complete your revisions, please reply to this message or contact the journal office at plosone@plos.org. When you’re ready to submit your revision, log on to https://www.editorialmanager.com/pone/ and select the 'Submissions Needing Revision' folder to locate your manuscript file.

Please include the following items when submitting your revised manuscript:

- A letter that responds to each point raised by the academic editor and reviewer(s). You should upload this letter as a separate file labeled 'Response to Reviewers'.
- A marked-up copy of your manuscript that highlights changes made to the original version. You should upload this as a separate file labeled 'Revised Manuscript with Track Changes'.
- An unmarked version of your revised paper without tracked changes. You should upload this as a separate file labeled 'Manuscript'.

If you would like to make changes to your financial disclosure, please include your updated statement in your cover letter. Guidelines for resubmitting your figure files are available below the reviewer comments at the end of this letter.

If applicable, we recommend that you deposit your laboratory protocols in protocols.io to enhance the reproducibility of your results. Protocols.io assigns your protocol its own identifier (DOI) so that it can be cited independently in the future. For instructions see: https://journals.plos.org/plosone/s/submission-guidelines#loc-laboratory-protocols. Additionally, PLOS ONE offers an option for publishing peer-reviewed Lab Protocol articles, which describe protocols hosted on protocols.io. Read more information on sharing protocols at https://plos.org/protocols?utm_medium=editorial-email&utm_source=authorletters&utm_campaign=protocols.

We look forward to receiving your revised manuscript.

Kind regards,

Janet E Rosenbaum, Ph.D.

Academic Editor

PLOS One

Journal Requirements:

When submitting your revision, we need you to address these additional requirements.

1. Please ensure that your manuscript meets PLOS ONE's style requirements, including those for file naming. The PLOS ONE style templates can be found at

https://journals.plos.org/plosone/s/file?id=wjVg/PLOSOne_formatting_sample_main_body.pdf and

https://journals.plos.org/plosone/s/file?id=ba62/PLOSOne_formatting_sample_title_authors_affiliations.pdf

2. Please note that your Data Availability Statement is currently missing the a direct link to access each database. If your manuscript is accepted for publication, you will be asked to provide these details on a very short timeline. We therefore suggest that you provide this information now, though we will not hold up the peer review process if you are unable.

3. Please include your full ethics statement in the ‘Methods’ section of your manuscript file. In your statement, please include the full name of the IRB or ethics committee who approved or waived your study, as well as whether or not you obtained informed written or verbal consent. If consent was waived for your study, please include this information in your statement as well.

4. If the reviewer comments include a recommendation to cite specific previously published works, please review and evaluate these publications to determine whether they are relevant and should be cited. There is no requirement to cite these works unless the editor has indicated otherwise.

[Note: HTML markup is below. Please do not edit.]

Reviewers' comments:

Reviewer's Responses to Questions

**Comments to the Author**

1. Is the manuscript technically sound, and do the data support the conclusions?

The manuscript must describe a technically sound piece of scientific research with data that supports the conclusions. Experiments must have been conducted rigorously, with appropriate controls, replication, and sample sizes. The conclusions must be drawn appropriately based on the data presented.

Reviewer #1: No

Reviewer #2: Yes

Reviewer #3: No

**********

2. Has the statistical analysis been performed appropriately and rigorously?

Reviewer #1: No

Reviewer #2: Yes

Reviewer #3: No

**********

3. Have the authors made all data underlying the findings in their manuscript fully available?

The PLOS Data policy requires authors to make all data underlying the findings described in their manuscript fully available without restriction, with rare exception (please refer to the Data Availability Statement in the manuscript PDF file). The data should be provided as part of the manuscript or its supporting information, or deposited to a public repository. For example, in addition to summary statistics, the data points behind means, medians and variance measures should be available. If there are restrictions on publicly sharing data—e.g. participant privacy or use of data from a third party—those must be specified.

Reviewer #1: No

Reviewer #2: Yes

Reviewer #3: No

**********

4. Is the manuscript presented in an intelligible fashion and written in standard English?

PLOS ONE does not copyedit accepted manuscripts, so the language in submitted articles must be clear, correct, and unambiguous. Any typographical or grammatical errors should be corrected at revision, so please note any specific errors here.

Reviewer #1: Yes

Reviewer #2: Yes

Reviewer #3: Yes

**********

5. Review Comments to the Author

Please use the space provided to explain your answers to the questions above. You may also include additional comments for the author, including concerns about dual publication, research ethics, or publication ethics. (Please upload your review as an attachment if it exceeds 20,000 characters)

Reviewer #1: Overall, the manuscript is presented poorly, and the described methodology is below par in quality. I highlight some of the discrepancies below. This manuscript needs major changes before it could reach the publication stage.

In the opening statement in the Introduction section (lines 46-47) I'd recommend insert the phrase "since the 1960s" for better clarity.

Expand the first occurrence of GPA on line 121

On line 105 the authors stated that the screening questionnaires were administered "....before a mass shooting and several months after." Obviously, the authors would not have predicted a MCE. Hence the description of this research study as "pre/post" (see line 159) appears to be somewhat misleading. It appears the initial data gathering step of this research process "pre" was done for a different reason. I'd recommend the authors provide some clarity on why the initial step of the screening was conducted, it's aims and objectives. Lines 116-118, the authors state: "...... along with a trigger warning to inform participants of the inclusion of 118 questions related to the on-campus shooting." I suspect this process pertains only to the second step of data gathering ("post"). I'd recommend the authors separate the two steps of data gathering and describe the purposes separately. On lines 115-116 the authors state: "The questionnaire was 116 distributed between April 12, and May 11, 2024." Which step of the research was distributed between these dates? When was the other step distributed? Also, please describe the date on which the tragic event had occurred. I suspect it took place near the student union on 12/6 (based on information from tables 1 and 2). If so, please state it in the text of the manuscript.

Given the relatively large proportion of 36 out of 198 (see line 180), it would help the readers to have information about how statistically different respondents who did not fully complete the surveys compared to respondents who fully completed the surveys (see lines 180-181). Moreover, did all 162 respondents who "fully completed the survey" did so on all the administered questionnaire items?

In table 1: there is an "unknown" category under "Perceived discrimination mean". Please explain what the authors meant by "unknown" here.

On lines 220-221: the authors state: "Furthermore, there was also a treatment for anxiety and depression by time interaction.....". Please explain the nature and duration of this "treatment" in detail. Did all the survey respondents received the same "treatment" for exactly same duration of time? When did the treatment start and when did it end?

On line 399 the authors themselves acknowledge that their study was "cross-sectional" in nature. However, throughout the manuscript the study design was highlighted as "pre/post" which essentially is longitudinal design. It would help the reviewers if the authors could clarify on aforementioned discrepancies.

Reviewer #2: This is a robust and needed study, regardless of the relatively small response number.

I found this interesting to read as it expanded my awareness of the impact of MCEs.

As you consider edits, there are initials which need definitions, and in my search did not always find the correct definition: eCRF, R, NAAN.

The use of figures and tables helps to support the findings. However, their number was a bit overwhelming. I found Figure 5 most meaningful. Please correct the labeling on figures 1, 2, 3, 4, and 6 for clarity. Table 1 requires review for punctuation corrections.

Initially, I thought participants completed two surveys: one before the MCE and one after – I was left wondering how one knew when “before” an MCE would be. Rather, this was a single survey of participant reflections upon their experiences before and after the MCE. Perhaps it’s more accurate that respondents reflected on their experiences prior to and after the MCE and completed a self-assessment survey.

Comparing facets of belonging, race, anxiety, depression, and race on a college campus to what supports individuals seek is important information regardless of experiencing an MCE. And yet crucial to knowing as universities prepare for such situations. Thank you for undertaking this important work.

Reviewer #3: This manuscript examines how students and staff at a minority-serving institution (MSI) were affected by a mass shooting event (MSE), with attention to changes in mental health, sense of belonging, and fitness. The manuscript also examined whether and how study participants sought help after the MSE. The topic is timely and important. There remains a notable gap in survey-based research that systematically examines the range of social, emotional, and psychological sequelae following MSEs. The authors’ focus on both students and staff is also potentially valuable, as staff experiences are often underexamined in this literature.

However, despite the importance of the topic, the manuscript currently has a number of conceptual and methodological weaknesses that limit its interpretability and contribution. Most critically, there is significant ambiguity surrounding the study design, timing of data collection relative to the MSE, and whether the data are longitudinal or cross-sectional. These issues, along with concerns about construct selection, analytic organization, and measurement reporting, must be addressed before the manuscript can make a meaningful contribution to the field. More details are provided below.

Major Concerns

The manuscript does not clearly specify whether participants were surveyed at two time points (pre- and post-MSE) or whether different participants were surveyed at different times. At several points, the language implies a pre–post design with the same participants, yet the limitations section describes the study as cross-sectional. This inconsistency creates substantial confusion and makes it difficult to interpret any claims about “change.”

If participants were not surveyed at two time points, this must be stated explicitly and consistently throughout the manuscript. A cross-sectional study, by definition, implies that participants were surveyed only once. Any language suggesting within-person change (e.g., pre–post effects) should be removed or revised accordingly. All language that implies causality, such as “impacted” should be removed. For instance, replace “impacts” with “is associated with”.

Critically, the timing of survey administration relative to the MSE must be made clear. Information about when baseline data were collected, when the MSE occurred, and when post-event data were collected should be summarized in the paragraph immediately preceding the Methods section and then described in detail within the Methods. The details can be done without specifying the date of the MSE (e.g., report “average days elapsed between MSE and post-test date” and use as a control ) should the authors want to blind the date of the MSE so that the institution can remain anonymous. Without this information, readers cannot assess the internal validity of the study or the plausibility of temporal interpretations.

Relatedly, the statistical approach should reflect the actual design. If the data are cross-sectional, the analyses must be framed accordingly, and the manuscript should include a clear discussion of sample size, group comparability, and the limitations of between-group comparisons over time.

A second major concern is that the manuscript reads as though this study may have been embedded within a larger research project that was already underway when the MSE occurred. If this is the case, it is essential to clarify why participants were being surveyed in the first place, what they were told about the study, and whether the MSE-related questions were added after the event.

Details about the broader study context—including recruitment procedures, study aims at the time of consent, and whether the MSE analysis was planned or opportunistic—should be explicitly described. This information is necessary for evaluating potential selection bias, response bias, and the interpretability of findings.

A third major concern is that the set of constructs examined in the manuscript lacks conceptual cohesion. While changes in mental health outcomes such as depression and anxiety are clearly relevant following an MSE, the inclusion of fitness is insufficiently justified. The manuscript does not make a compelling theoretical or empirical case for why fitness should be examined as a primary outcome alongside mental health and belonging. The paper would be substantially strengthened by focusing on psychological functioning (e.g., depression, anxiety, distress) as the primary outcomes, with constructs such as belonging and fitness examined as moderators or mediators. This approach would improve conceptual clarity and align the analyses more closely with existing trauma and stress literature.

Similarly, the inclusion of the research question related to help-seeking behavior after the MSE appears underdeveloped and somewhat tangential. This research question is not well integrated into the manuscript’s theoretical framework and reads as an afterthought—and is also too much material/too many topics to cover with the care they need. The authors may wish to consider focusing this paper on psychological and social outcomes following the MSE and reserving help-seeking analyses for a separate manuscript.

Fourth, while the manuscript provides solid information regarding the reliability and validity of the belonging measure, similar information is missing or insufficient for other key measures. For all scales used, the authors should report as much information about reliability and briefly describe evidence of prior validity and note any potential areas where measures are new or not yet fully validated and validated with a minority sample. Given the centrality of measurement quality to the manuscript’s claims, this information is essential.

Less Major Concerns

The sample includes both students and staff, who are likely to differ substantially in exposure, role-related stressors, access to resources, and help-seeking norms. This reviewer suggests that student versus staff status should be examined explicitly as a moderator. Doing so would allow for more nuanced interpretation and would strengthen the manuscript’s contribution by carefully identifying and discussing whether the effects of the MSE differ across institutional roles. This is also another reason why an examination of help-seeking should not be included in this manuscript, as the help-seeking paths (and potential pathways) and reasons will greatly differ across students versus adult employees who are not students.

An topic that needs more clarity is related to the IRB review. The manuscript notes that the study was considered IRB-exempt, but provides no explanation for this determination. Given that the study involves human subjects and aims to produce generalizable knowledge, further clarification is needed. The authors should explain the basis for the exemption (e.g., anonymous data collection, minimal risk, use of existing data). Providing this information would increase transparency and reassure readers that ethical considerations were appropriately addressed—particularly important given the sensitive nature of the topic (even with anonymity promised).

Last, the results section is difficult to follow, as findings related to different constructs are interwoven without a clear analytic structure. A more coherent approach would be to first present results related to changes in mental health outcomes, followed by analyses examining moderators or mediators (e.g., belonging, fitness, staff vs. student status). Reorganizing the results section in this way would greatly enhance clarity and help readers understand what was found and for whom.

**********

6. PLOS authors have the option to publish the peer review history of their article (what does this mean?). If published, this will include your full peer review and any attached files.

If you choose “no”, your identity will remain anonymous but your review may still be made public.

**Do you want your identity to be public for this peer review?**  For information about this choice, including consent withdrawal, please see our Privacy Policy.

Reviewer #1: **Yes:**  Lakshminarayana Chekuri, MD, PhD

Reviewer #2: No

Reviewer #3: **Yes:**  Caterina G. Roman

**********

[NOTE: If reviewer comments were submitted as an attachment file, they will be attached to this email and accessible via the submission site. Please log into your account, locate the manuscript record, and check for the action link "View Attachments". If this link does not appear, there are no attachment files.]

To ensure your figures meet our technical requirements, please review our figure guidelines: https://journals.plos.org/plosone/s/figures

You may also use PLOS’s free figure tool, NAAS, to help you prepare publication quality figures: https://journals.plos.org/plosone/s/figures#loc-tools-for-figure-preparation.

NAAS will assess whether your figures meet our technical requirements by comparing each figure against our figure specifications.

# Author comment: Author response to Decision Letter 1

**DOI:** 10.1371/journal.pone.0353453.r002

16 Feb 2026

1. Please ensure that your manuscript meets PLOS ONE's style requirements, including those for file naming.

Thank you for the comment. We have reviewed the PLOS ONE style requirements and modified all areas where formatting and naming conventions so they are now aligned with the journal.

2. Please note that your Data Availability Statement is currently missing the a direct link to access each database. If your manuscript is accepted for publication, you will be asked to provide these details on a very short timeline. We therefore suggest that you provide this information now, though we will not hold up the peer review process if you are unable.

Thank you for the comment. We have added a link to the open science framework repository that contains the data and code for this manuscript. The link is currently set to view only, but will be released publicly upon acceptance.

https://osf.io/5ad62/overview?view_only=d36c33d0ebf943aeaf7ea75e29d52432

3. Please include your full ethics statement in the ‘Methods’ section of your manuscript file. In your statement, please include the full name of the IRB or ethics committee who approved or waived your study, as well as whether or not you obtained informed written or verbal consent. If consent was waived for your study, please include this information in your statement as well.

Thank you for the comment, we have included the university ethics approval and consent process specific to this study.

This information was added in the Methods section. (Revised text below).

The study was exempted by the Office of Research Integrity – Social Behavioral Institutional Review Board of the University of Nevada, Las Vegas (protocol code #UNLV-2024-129 – February 20, 2024). The study was classified as Category 2(i) as the information obtained was recorded by the investigator in such a manner that the identity of the human subjects cannot readily be ascertained, directly or through identifiers linked to the subjects. Informed consent was provided at the start of the online questionnaire, along with a trigger warning to inform participants of the inclusion of questions related to the on-campus shooting, which occurred approximately 6 months prior to survey distribution. Upon acceptance of the electronic informed consent, the self-administered survey collected demographic information including status (employee or student), grade point average (GPA) for students only, age, race, ethnicity, sex, gender identity, sexual orientation, height, and weight.

Response to Reviewer Comments

Reviewer #1: Overall, the manuscript is presented poorly, and the described methodology is below par in quality. I highlight some of the discrepancies below. This manuscript needs major changes before it could reach the publication stage.

Thank you for the opportunity to clarify our methodology and revise this manuscript for publication. Much has been revised to improve clarity and thanks to reviewer recommendations, we believe the work is much improved.

Reviewer 1; Comment 1: In the opening statement in the Introduction section (lines 46-47) I'd recommend insert the phrase "since the 1960s" for better clarity.

Thank you for the comment. We clarity of message is important to us. The sentence was rewritten for better clarity.

Propose change to manuscript based on comment:

From the 1960’s to the end of 2023, thirteen college campuses in the United States were targets of mass shootings resulting in 102 deaths, as well as numerous physical, and emotional injuries[1].

Reviewer 1; Comment 2: Expand the first occurrence of GPA on line 121.

Thank you for catching this. We have expanded GPA at its first occurrence.

Reviewer 1; Comment 3: On line 105 the authors stated that the screening questionnaires were administered "....before a mass shooting and several months after." Obviously, the authors would not have predicted a MCE. Hence the description of this research study as "pre/post" (see line 159) appears to be somewhat misleading. It appears the initial data gathering step of this research process "pre" was done for a different reason. I'd recommend the authors provide some clarity on why the initial step of the screening was conducted, it's aims and objectives.

Lines 116-118, the authors state: "...... along with a trigger warning to inform participants of the inclusion of 118 questions related to the on-campus shooting." I suspect this process pertains only to the second step of data gathering ("post"). I'd recommend the authors separate the two steps of data gathering and describe the purposes separately. On lines 115-116 the authors state: "The questionnaire was 116 distributed between April 12, and May 11, 2024." Which step of the research was distributed between these dates? When was the other step distributed? Also, please describe the date on which the tragic event had occurred. I suspect it took place near the student union on 12/6 (based on information from tables 1 and 2). If so, please state it in the text of the manuscript.

REPONSE TO ADDRESS DISCREPANCY IN SURVEY TIMELINE (reference for all reviewers)

Thank you for making us aware of this major oversight, which was raised consistently by all reviewers. In response, and based on the importance of this feedback, we have made significant revisions throughout the manuscript to improve clarity, transparency, and alignment between the study purpose, methods, and interpretation of findings. These changes extend beyond a single section and were implemented across the abstract, introduction, methods, results, and discussion to ensure that the scope of the study and the rationale for included variables are clearly articulated. We believe these revisions substantially clarify the study methodology, strengthen the manuscript and address the reviewers’ concerns. We regret that the survey design and reporting time frames were not described with sufficient clarity in the original submission, and we appreciate the reviewers’ comments.

In short, the complete survey was distributed once, after the MCE had occurred. Within the survey the PHQ-9 and GAD-7 are delivered twice, with each instance having a unique prompt for participants to recall their feelings before the MCE and after the MCE. When we originally presented survey scores as “pre-event” that was incorrect. The survey was not distributed prior to the event. Rather we asked participants to recall their pre-event feelings for the first instance of the PHQ-9 and GAD-7 and then recall them again as they feel currently, after the event.

We have revised language throughout the manuscript to clearly indicate that this was a cross-sectional survey focused on recall of past feelings, rather than a longitudinal study that distributed the survey pre and post MCE. The survey was administered between April 12, and May 11, 2024 after the MCE which occurred on December 6, 2023. And again, participants were asked to recall their pre-MCE feelings first and then recall their current (post-MCE) feelings. Differences in these different recall types were the focus of our analysis.

Specifically, participants were first asked to recall their state of mind from approximately six months before the mass casualty event, and complete PHQ-9 & GAD-7. The participants then reported their feelings a second time with the prompt to report their current level of depression through the PHQ-9 and anxiety as evaluated by the GAD-7.

As each reviewer has correctly pointed out, there were instances in the manuscript where these two timeframes were referred to as ‘pre’ and ‘post,’ which erroneously led readers to think that participants completed the PHQ-9 and GAD-7 at two different times. To clarify the timeline of this study and it’s reporting in the results the timeframes have been renamed, Pre-MCE Recall and Recent Recall to better describe when the survey was taken and what the outcomes of the survey represent.

Reviewer 1; Comment 4: Given the relatively large proportion of 36 out of 198 (see line 180), it would help the readers to have information about how statistically different respondents who did not fully complete the surveys compared to respondents who fully completed the surveys (see lines 180-181). Moreover, did all 162 respondents who "fully completed the survey" did so on all the administered questionnaire items?

In table 1: there is an "unknown" category under "Perceived discrimination mean". Please explain what the authors meant by "unknown" here.

Thank you for the comment and catching the “unknown” designation for the PEDQ questionnaire as reported in Table 1. These five individuals are listed as unknown because they did not complete that portion of the survey. These individuals have been removed from the data set (as seen in Table) and all analyses have been updated to reflect that change. Their removal had no effect on the primary and secondary results of the study.

To address your initial comment regarding possible demographic or other survey responses differences between completers and non-completers of the survey, 36 of the 41 participants did not respond beyond recording their role at UNLV (student, student and employee, or employee only). Therefore, we performed an analysis looking at how role at UNLV was associated with completion or non-completion of the study and observed no relationship. Among the 5 participants who completed more than their role at UNLV, but not the entire survey, each only partially completed the PEDQ. These five participants were similar to the completers of the survey, which leaves us with limited information on what about this questionnaire may have caused their limited response.

Proposed change in manuscript in response to comment (lines 213-218)

“Logistic regression was used to examine any relationship between those who completed or did not fully complete the survey, with survey completeness (yes/no) as the outcome variable and role at the university as the predictor variable. Summary statistics of those who partially complete the survey were calculated and compared to the sample carried forward to describe potential bias in partial completion. “

“Completer analysis demonstrated that those who completed the survey versus those who did not complete the survey was not related to their role at the university (χ(2) = 3.94, p = 0.14). Five participants did complete a portion of the survey beyond reporting of their initial role. Among these partial completers, each reported similar demographic and survey responses like those who fully completed the survey. Interestingly, each did not fully complete the PEDQ, but we have no information on why this questionnaire within the survey was left incomplete among these participants.”

Reviewer 1; Comment 5: On lines 220-221: the authors state: "Furthermore, there was also a treatment for anxiety and depression by time interaction.....". Please explain the nature and duration of this "treatment" in detail. Did all the survey respondents received the same "treatment" for exactly same duration of time? When did the treatment start and when did it end?

The question, “Are you currently taking medication for or participating in counseling or psychotherapy for the treatment of depression or anxiety?” is further specified on lines 142–143 and was intended to assess individual participation in medication use or therapy. No intervention was performed as part of this study.

Updated text in the manuscript methods

“Participants were asked whether they were currently using antidepressant medication or engaged in counseling or psychotherapy for depression treatment.”

Reviewer 1; Comment 6: On line 399 the authors themselves acknowledge that their study was "cross-sectional" in nature. However, throughout the manuscript the study design was highlighted as "pre/post" which essentially is longitudinal design. It would help the reviewers if the authors could clarify on aforementioned discrepancies.

Thank you for the comment and attention to detail on this point. Please see our complete response to this discrepancy as you have brought in your earlier comment. The authors appreciate the reviewer’s feedback and regret the lack of clarity on this point in the original submission. This was a cross-sectional study intended to provide insight into factors that may support or hinder campus recovery following a mass shooting, an area in which the existing research remains limited.

Reviewer #2: This is a robust and needed study, regardless of the relatively small response number. I found this interesting to read as it expanded my awareness of the impact of MCEs.

As you consider edits, there are initials which need definitions, and in my search did not always find the correct definition: eCRF, R, NAAN.

Thank you for this comment. We have reviewed the manuscript to ensure that abbreviations are used correctly and have limited their use where possible. eCRF is for Estimated Cardiorespiratory Fitness, R is for the statistical programming language R, and NAAN is for Native Alaskan/American Indian.

Reviewer 2; Comment 1: The use of figures and tables helps to support the findings. However, their number was a bit overwhelming. I found Figure 5 most meaningful. Please correct the labeling on figures 1, 2, 3, 4, and 6 for clarity. Table 1 requires review for punctuation corrections.

Thank you for the comment and we appreciate the keen attention to detail in presentation of results. We have combined figures to now have 5 in total and have removed the model result tables to provide a cleaner and better reading experience. Rest assured that all model results can be viewed in the open science framework repository that the data and code that produced the results will be stored, if prospective readers are interested in the granular level outcomes of this research.

Reviewer 2; Comment 1: Initially, I thought participants completed two surveys: one before the MCE and one after – I was left wondering how one knew when “before” an MCE would be. Rather, this was a single survey of participant reflections upon their experiences before and after the MCE. Perhaps it’s more accurate that respondents reflected on their experiences prior to and after the MCE and completed a self-assessment survey.

Thank you for the comment and keen attention to detail in this regard. It is an area brought up by each reviewer. We have addressed this comment at length in an early comment by Reviewer 1, please see above, and we invite you to read that comment for the complete response. In short, data collection occurred between April 12 and May 11, and participants were asked to recall their state of mind prior to the shooting as well as during the two weeks preceding survey completion. We regret the lack of clarity in the original submission and have addressed this concern through substantial revisions throughout the manuscript. We have now shifted language throughout the manuscript to reflect that measures of PHQ-9 and GAD7 are of participant recall before and after the MCE, and not that these surveys were delivered before and after the MCE.

Reviewer 2; Comment 2: Comparing facets of belonging, race, anxiety, depression, and race on a college campus to what supports individuals seek is important information regardless of experiencing an MCE. And yet crucial to knowing as universities prepare for such situations. Thank you for undertaking this important work.

We would like to express our deep gratitude for this comment. It is our hope that better understand of campus resilience and areas of need after such unfortunate events can help in response and recovery for future events.

Reviewer #3: This manuscript examines how students and staff at a minority-serving institution (MSI) were affected by a mass shooting event (MSE), with attention to changes in mental health, sense of belonging, and fitness. The manuscript also examined whether and how study participants sought help after the MSE. The topic is timely and important. There remains a notable gap in survey-based research that systematically examines the range of social, emotional, and psychological sequelae following MSEs. The authors’ focus on both students and staff is also potentially valuable, as staff experiences are often underexamined in this literature.

However, despit

Attachment Submitted filename: Response to Reviewers.docx

# Peer review: Decision Letter 1

**DOI:** 10.1371/journal.pone.0353453.r003

**Authors:** Janet Rosenbaum, Janet Rosenbaum

14 Apr 2026

PONE-D-25-33879R1

Supporting recovery: Intersections of health, belonging, and help seeking after a campus mass casualty event

PLOS One

Dear Dr. Jalene,

Thank you for submitting your manuscript to PLOS ONE. After careful consideration, we feel that it has merit but does not fully meet PLOS ONE’s publication criteria as it currently stands. Therefore, we invite you to submit a revised version of the manuscript that addresses the points raised during the review process.

In particular, one reviewer thought that the recall bias was substantial because respondents were asked to recall their psychological state prior to the mass shooting event, and that this limitation should be noted prominently and discussed. Further, the measures should be described appropriately to explicitly note the recall in each case as "recalled pre-shooting" or "recalled baseline" as opposed to a baseline. You need to cite research that validates your approach to administering the PHQ-9 and GAD-7 in succession, first about recalled pre-shooting and second about the past 2 weeks. As the reviewer said, the recollections of emotions prior to a traumatic event are likely to have been reported differentially based on current psychological state.

Please submit your revised manuscript by May 29 2026 11:59PM. If you will need more time than this to complete your revisions, please reply to this message or contact the journal office at plosone@plos.org. When you're ready to submit your revision, log on to https://www.editorialmanager.com/pone/ and select the 'Submissions Needing Revision' folder to locate your manuscript file.

Please include the following items when submitting your revised manuscript:

- A letter that responds to each point raised by the academic editor and reviewer(s). You should upload this letter as a separate file labeled 'Response to Reviewers'.
- A marked-up copy of your manuscript that highlights changes made to the original version. You should upload this as a separate file labeled 'Revised Manuscript with Track Changes'.
- An unmarked version of your revised paper without tracked changes. You should upload this as a separate file labeled 'Manuscript'.

If you would like to make changes to your financial disclosure, please include your updated statement in your cover letter. Guidelines for resubmitting your figure files are available below the reviewer comments at the end of this letter.

If applicable, we recommend that you deposit your laboratory protocols in protocols.io to enhance the reproducibility of your results. Protocols.io assigns your protocol its own identifier (DOI) so that it can be cited independently in the future. For instructions see: https://journals.plos.org/plosone/s/submission-guidelines#loc-laboratory-protocols. Additionally, PLOS ONE offers an option for publishing peer-reviewed Lab Protocol articles, which describe protocols hosted on protocols.io. Read more information on sharing protocols at https://plos.org/protocols?utm_medium=editorial-email&utm_source=authorletters&utm_campaign=protocols.

As the corresponding author, your ORCID iD is verified in the submission system and will appear in the published article. PLOS supports the use of ORCID, and we encourage all coauthors to register for an ORCID iD and use it as well. Please encourage your coauthors to verify their ORCID iD within the submission system before final acceptance, as unverified ORCID iDs will not appear in the published article. *Only*  the individual author can complete the verification step; PLOS staff *cannot*  verify ORCID iDs on behalf of authors.

We look forward to receiving your revised manuscript.

Kind regards,

Janet E Rosenbaum, Ph.D.

Academic Editor

PLOS One

**Journal Requirements:**

If the reviewer comments include a recommendation to cite specific previously published works, please review and evaluate these publications to determine whether they are relevant and should be cited. There is no requirement to cite these works unless the editor has indicated otherwise.

[Note: HTML markup is below. Please do not edit.]

Reviewers' comments:

Reviewer's Responses to Questions

**Comments to the Author**

1. If the authors have adequately addressed your comments raised in a previous round of review and you feel that this manuscript is now acceptable for publication, you may indicate that here to bypass the “Comments to the Author” section, enter your conflict of interest statement in the “Confidential to Editor” section, and submit your "Accept" recommendation.

Reviewer #2: All comments have been addressed

Reviewer #3: (No Response)

**********

2. Is the manuscript technically sound, and do the data support the conclusions?

The manuscript must describe a technically sound piece of scientific research with data that supports the conclusions. Experiments must have been conducted rigorously, with appropriate controls, replication, and sample sizes. The conclusions must be drawn appropriately based on the data presented.

Reviewer #2: Yes

Reviewer #3: No

**********

3. Has the statistical analysis been performed appropriately and rigorously?

Reviewer #2: Yes

Reviewer #3: No

**********

4. Have the authors made all data underlying the findings in their manuscript fully available?

The PLOS Data policy requires authors to make all data underlying the findings described in their manuscript fully available without restriction, with rare exception (please refer to the Data Availability Statement in the manuscript PDF file). The data should be provided as part of the manuscript or its supporting information, or deposited to a public repository. For example, in addition to summary statistics, the data points behind means, medians and variance measures should be available. If there are restrictions on publicly sharing data—e.g. participant privacy or use of data from a third party—those must be specified.

Reviewer #2: Yes

Reviewer #3: Yes

**********

5. Is the manuscript presented in an intelligible fashion and written in standard English?

PLOS ONE does not copyedit accepted manuscripts, so the language in submitted articles must be clear, correct, and unambiguous. Any typographical or grammatical errors should be corrected at revision, so please note any specific errors here.

Reviewer #2: Yes

Reviewer #3: Yes

**********

6. Review Comments to the Author

Please use the space provided to explain your answers to the questions above. You may also include additional comments for the author, including concerns about dual publication, research ethics, or publication ethics. (Please upload your review as an attachment if it exceeds 20,000 characters)

Reviewer #2: I appreciate your thorough response to 3 reviewers’ comments and the attention to revising the manuscript to increase readability and transparency. In this second reading of the manuscript, I am reminded that there are multiple authors, and each one has a voice. Below in my later comments I identify clear language used which might inform your responses to preceding comments. Thank you for considering this feedback:

Line 36-37 In the abstract, “Increases in depression were linked to lower pre-MCE anxiety, higher PEDQ-CV scores, and receiving current treatment for mental health concerns.” So those who had low anxiety prior to the MCE, overall higher perceived discrimination AND receiving current treatment had an increase in depression post-MCE? Please confirm how are these three things are related to an increase in depression.

Lines 71: Editorial suggestion to cut “there are” for increased readability: “The trauma of exposure to an active shooter and loss of life in one’s normal living environment may exacerbate these issues,[2]and other factors may help or hurt the recovery process.”

Line 72: Editorial suggestion to cut “also” for increased readability: “Low fitness is associated with poorer mental health outcomes[14]…”

Line 110: In the prior line, you mention retrospectively reporting symptoms, but in 110 do not specific the length of the period prior to the mass shooting. “…from the period prior…” tho you go on to specify “during the two weeks immediately preceding survey completion” in line 111. If you’re going to specify one time period, please specify the other.

Line 112: You identify “survey instruments” but because this is not pre/post, it’s ONE long survey incorporating multiple measurements, yes? Asking folks to reflect twice on their symptoms, before the MCE and after. Please clarify that you have incorporated multiple questionnaires into one self-report survey. (Line 263 uses “questionnaire within the survey” which could provide clarity in line 112).

Line 121: Appreciate the additional information in this section, however not every reader will understand what a “Category 2(i)” is. At my university, we would call the description that followed as “Exempt.” Perhaps adding that term in parentheses would be a sufficient explanation.

Lines 136-141: Although an improved survey description, it still sounds like these measurements/instruments are being administered separately, rather than the self-administered survey, it two parts, included/repeated such measurements : for the X time period retrospective, and then the more immediate reflection of after the MCE. Or somesuch. This section needs additional clarity. Note: subsequently, you describe the PHQ-9, GAD-7, SBS (line 164), PEDQ (line 176), CRF (line 185), and PA-I (line 193), however you do not include the last 4 items in lines 136-141. If you are using this section to summarize the survey, please include all instruments/questionaires incorporated into the single survey for clarity.

Line 185: You lost me. Is the CRF estimation part of your analysis or self-reported data collection? It looks like the PA-1 and POSSIBLY the RHR was collected as part of the self-report survey. Please clarify what was collected in the survey prior to diving into how the information was analyzed… Please clarify if you used self-report information to generate the eCRF.

Lines 208-209: “subsequent analyses” re: eCRF are references here but I don’t see them in the statistical analyses and minimally in the results section (Lines 343, 385, solely related to employees).

Line 229: Reference to “(pre/post)” to be removed and clarified to avoid reader confusion. At one point, 6 months of recall vs. 2 weeks immediately prior to the survey is mentioned earlier in the manuscript. For consistency, repeat those time frames throughout the study to remind the reader to the time frames participants were to recall.

Lines 227-234: This is a long section that begins with identifying independent variable, covariates, and ends with “were used as control variables.” Is there a period missing somewhere? Please clarify that which were control variables and that which were not.

Line 235 is the clearest explanation of how the survey was presented “between recall (pre-MCE verses current)” – consider using this explainer throughout.

Line 263: Again, the clearest explanation of a measurement within the survey – consider using throughout: “questionnaire within the survey”

Line 273: Table 1 Participant Demographics. You have included measurement responses along with participant demographics. And even then, you do not include ALL of the measurement response. Perhaps separate the information between simple demographics and questionnaire response means.

Line 276: BRAVA! “…pre MCE and recent recall” is the best descriptor I’ve read yet! See if this language can be incorporated throughout for increased clarity.

Line 285: Yes! “Recall difference” makes your research so much clearer. Thank you!

Line 301-302: Increased clarity needed. Perhaps just restating the previous sentence about the relationship between pre-MCE recall GAD-7 and both recall time points for PHQ-9. (Or perhaps I will better understand the results section when I get to the discussion section.) ;)

Line 309: Typo? “Furthermore there was also a treatment…” Instead of treatment, interaction?

Line 315: Replace “pre/post”

Line 392-393: This feels important to restate “…employees who reported higher perceived discrimination also had higher [anxiety]…”

Lines 440-446: This feels like a significant finding. Consider adding the information from lines 392-393.

Lines 463-471: discuss the importance of physical activity on mental health but lacks specific information from respondents. There is no discussion about eCRF, BMI, or the PA-I information gleaned from participants. Regardless if cardiovascular fitness is important to participants, please include this in the discussion. Fitness levels of participants were not part of the discussion.

This manuscript is getting closer to publication. I recommend addressing these revisions prior to resubmitting. This is an important study! Thank you for the opportunity to read your revised manuscript.

Reviewer #3: Although the authors acknowledged the cross sectional design, a central remaining concern involves the use of retrospective self-reports to approximate pre-event functioning within what is now clarified to be a cross-sectional design. While the authors have improved clarity around the study design, the analytic approach still relies on participants’ recall of their pre-MSE mental health, belonging, and related states to draw inferences about change. This approach raises significant concerns about measurement validity.

A large body of methodological and psychological research has demonstrated that retrospective reports of prior states—especially following traumatic or highly salient events such as a mass shooting—are systematically biased (see for instance review in Faul & LaBar, 2023). Recall bias, mood-congruent memory, and cognitive reconstruction processes can lead individuals to reinterpret their past functioning in light of their current experiences. Basically, people recall past experiences in ways that are consistent with their current mood. For example, individuals experiencing distress post-event may recall their pre-event functioning as better than it actually was, thereby inflating perceived declines; alternatively, some may normalize past distress, attenuating perceived change. These processes are well-documented and pose a direct threat to the validity of inferred pre–post differences when based on retrospective recall. Furthermore, for people have experienced depression or who are currently depressed, recall may be even less reliable and valid measures. See for instance:Brand et al., 1992 in Journal of Affective Disorders.

Accordingly, the “pre” measures in this study should not be treated as equivalent to true baseline data, and the resulting comparisons cannot be interpreted as estimates of within-person change. At most, they capture perceived or reconstructed change, which is conceptually distinct. This reviewer notes that the authors do utilize the term "perceived" change in this revision, but nonetheless, it remains very difficult to interpret what the change signifies and hence the implications of these finings. Basically, this distinction has important implications for how the findings are framed and interpreted. The manuscript would be strengthened by explicitly acknowledging these limitations in the relevant places (methods, limitations and conclusion).

Faul, L., & LaBar, K. S. (2023). Mood-congruent memory revisited. Psychological review, 130(6), 1421–1456. https://doi.org/10.1037/rev0000394

**********

7. PLOS authors have the option to publish the peer review history of their article (what does this mean?). If published, this will include your full peer review and any attached files.

If you choose “no”, your identity will remain anonymous but your review may still be made public.

**Do you want your identity to be public for this peer review?**  For information about this choice, including consent withdrawal, please see our Privacy Policy.

Reviewer #2: No

Reviewer #3: **Yes:**  Caterina Roman

**********

[NOTE: If reviewer comments were submitted as an attachment file, they will be attached to this email and accessible via the submission site. Please log into your account, locate the manuscript record, and check for the action link "View Attachments". If this link does not appear, there are no attachment files.]

To ensure your figures meet our technical requirements, please review our figure guidelines: https://journals.plos.org/plosone/s/figures

You may also use PLOS’s free figure tool, NAAS, to help you prepare publication quality figures: https://journals.plos.org/plosone/s/figures#loc-tools-for-figure-preparation.

NAAS will assess whether your figures meet our technical requirements by comparing each figure against our figure specifications.

# Author comment: Author response to Decision Letter 2

**DOI:** 10.1371/journal.pone.0353453.r004

1 May 2026

Review Comments to the Author

Please use the space provided to explain your answers to the questions above. You may also include additional comments for the author, including concerns about dual publication, research ethics, or publication ethics. (Please upload your review as an attachment if it exceeds 20,000 characters)

Reviewer #2: I appreciate your thorough response to 3 reviewers’ comments and the attention to revising the manuscript to increase readability and transparency. In this second reading of the manuscript, I am reminded that there are multiple authors, and each one has a voice. Below in my later comments I identify clear language used which might inform your responses to preceding comments. Thank you for considering this feedback:

Line 36-37 In the abstract, “Increases in depression were linked to lower pre-MCE anxiety, higher PEDQ-CV scores, and receiving current treatment for mental health concerns.” So those who had low anxiety prior to the MCE, overall higher perceived discrimination AND receiving current treatment had an increase in depression post-MCE? Please confirm how are these three things are related to an increase in depression.

Thank you for the comment and sincere feedback! We have modified the reporting of the results in the abstract to be more clear on the direction of each variable on our measured outcome of depression. Below is the revised text in the abstract to clarify the link.

Increases in depression (PHQ-9 scores) from pre-MCE to recent recall were larger among respondents with lower pre-MCE anxiety (GAD-7 scores), higher perceived discrimination (PEDQ), and those currently receiving treatment for anxiety or depression; respondents not receiving treatment showed no significant change.

Lines 71: Editorial suggestion to cut “there are” for increased readability: “The trauma of exposure to an active shooter and loss of life in one’s normal living environment may exacerbate these issues,[2]and other factors may help or hurt the recovery process.”

Line 72: Editorial suggestion to cut “also” for increased readability: “Low fitness is associated with poorer mental health outcomes[14]…”

Thank you for the suggestion! We have updated the text accordingly.

Line 110: In the prior line, you mention retrospectively reporting symptoms, but in 110 do not specific the length of the period prior to the mass shooting. “…from the period prior…” tho you go on to specify “during the two weeks immediately preceding survey completion” in line 111. If you’re going to specify one time period, please specify the other.

Thank you for the comment! We have specified the recall time frame, which was (1) the two weeks immediately preceding the mass casualty event (approximately 4–5 months prior to survey administration), and (2) the two weeks immediately preceding completion of the questionnaire.

Line 112: You identify “survey instruments” but because this is not pre/post, it’s ONE long survey incorporating multiple measurements, yes? Asking folks to reflect twice on their symptoms, before the MCE and after. Please clarify that you have incorporated multiple questionnaires into one self-report survey. (Line 263 uses “questionnaire within the survey” which could provide clarity in line 112).

Thank you for this insight, indeed this was not clear. We have revised the text throughout the manuscript to detail the nature by which and when each measurement was collected.

Line 121: Appreciate the additional information in this section, however not every reader will understand what a “Category 2(i)” is. At my university, we would call the description that followed as “Exempt.” Perhaps adding that term in parentheses would be a sufficient explanation.

Thank you for this comment! We have updated the manuscript accordingly. Please see below for updated text.

The study was classified as exempt (Category 2(i) under 45 CFR 46.104(d)(2)) as the information obtained was recorded by the investigator in such a manner that the identity of the human subjects cannot readily be ascertained, directly or through identifiers linked to the subjects.).

Lines 136-141: Although an improved survey description, it still sounds like these measurements/instruments are being administered separately, rather than the self-administered survey, it two parts, included/repeated such measurements : for the X time period retrospective, and then the more immediate reflection of after the MCE. Or somesuch. This section needs additional clarity. Note: subsequently, you describe the PHQ-9, GAD-7, SBS (line 164), PEDQ (line 176), CRF (line 185), and PA-I (line 193), however you do not include the last 4 items in lines 136-141. If you are using this section to summarize the survey, please include all instruments/questionaires incorporated into the single survey for clarity.

This was an unfortunate oversight and your comments have prompted us to update the language, which will clarify the multiple components of the singular questionnaire. The entire passage has been updated. Please see below for updated text.

Upon providing electronic informed consent, participants completed a multi-faceted, self-administered questionnaire that included several validated instruments, including the Patient Health Questionnaire-9 (PHQ-9)[34] and the Generalized Anxiety Disorder-7 (GAD-7)[35]. Notably, the PHQ-9 and GAD-7 were the only measures requiring retrospective recall. For these instruments, participants reported their mood during two time periods: (1) the two weeks immediately preceding the mass casualty event (approximately 4–5 months prior to survey administration), and (2) the two weeks immediately preceding completion of the questionnaire. All other measures included in the questionnaire, including the Student Belongingness Survey (SBS)[36] and the Perceived Ethnic Discrimination Questionnaire (PEDQ)[32], assessed participants’ current state only.

Participants were also asked for demographic information, including university status (employee or student), grade point average (GPA; students only), age, race, ethnicity, sex, gender identity, sexual orientation, height, and weight. Body mass index (BMI) was calculated from self-reported height and weight using the formula weight (kg) / [height (m)]². A Physical Activity Index (PA-I) and estimated cardiorespiratory fitness (eCRF) were assessed using validated algorithms[33,37]. Participants reported whether they were currently using antidepressant medication or engaged in counseling or psychotherapy for depression treatment. Finally, participants stated their willingness to attend counseling sessions at the university (yes/no) and their interest in learning about alternative approaches, such as meditation and exercise (yes/no).

Line 185: You lost me. Is the CRF estimation part of your analysis or self-reported data collection? It looks like the PA-1 and POSSIBLY the RHR was collected as part of the self-report survey. Please clarify what was collected in the survey prior to diving into how the information was analyzed… Please clarify if you used self-report information to generate the eCRF.

Thank you for your comment and the opportunity to clarify! The algorithm used to calculate eCRF incorporates the PA-I and is described in greater detail in the Methods section. To improve clarity, we have revised the first mention in the Methods. The updated text is provided below:

A Physical Activity Index (PA-I) and estimated cardiorespiratory fitness (eCRF) were derived from participant data reported in the survey which are entered into validated algorithms for calculations (see further details below) [33,37].

…Cardiorespiratory fitness was estimated using a validated and reliable algorithm to examine responses to the trauma of the mass shooting event in relation to fitness level. Cardiorespiratory fitness reflects the cumulative effect of sedentary and physical activity behaviors. The eCRF measure developed by Nes et al. has demonstrated strong validity and has been shown to be comparable in accuracy to many field-based assessments of peak oxygen consumption (VO₂peak) [33,37]. The model was derived from cross-validation VO₂peak data from 4,367 healthy adults and has demonstrated acceptable accuracy for estimating sex-specific VO₂peak in outpatient and population-based settings[33,37]. The regression model includes age, body mass index (BMI), resting heart rate (RHR), and a physical activity index (PA-I). The PA-I was calculated from self-reported exercise frequency, duration, and intensity using a previously validated scoring system [33,37,39] and has shown moderate correlations with measured VO₂peak (r = 0.44 for men; r = 0.38 for women). Although the original model incorporated waist circumference, a BMI-based algorithm has been shown to produce comparable estimates [37]. Given inconsistencies in self-reported waist circumference, the BMI-based model was used in the present study.

Sex-specific equations were applied as follows:

Men (R² = 0.59, SEE = 5.8): 92.05 − (0.327 × age) − (0.933 × BMI) − (0.167 × RHR) + (0.257 × PA-I)

Women (R² = 0.57, SEE = 5.1): 70.77 − (0.244 × age) − (0.749 × BMI) − (0.107 × RHR) + (0.213 × PA-I)

For each participant, estimated CRF (eCRF) was compared with age-predicted normative CRF values for healthy adults[33,40]. To enhance methodological rigor, eCRF estimates and normative values were cross-verified using the web-based calculator developed by the original authors. Both eCRF and the difference between individual eCRF and normative CRF values (DIFF) were included in subsequent analyses.

Lines 208-209: “subsequent analyses” re: eCRF are references here but I don’t see them in the statistical analyses and minimally in the results section (Lines 343, 385, solely related to employees).

Thank you for this important observation! The reviewer is correct that, in the prior version, eCRF and the eCRF–normative difference (DIFF) were introduced in the Methods as variables to be carried into “subsequent analyses,” but the corresponding results were not consistently reported. We have addressed this in two ways. First, we standardized terminology so that eCRF and DIFF are referred to consistently throughout the Methods, Statistical Analysis, and Results, including explicit listing of cardiorespiratory fitness in the all-respondent, student sub-group, and employee sub-group analyses. Second, we added explicit reporting of cardiorespiratory fitness in the all-respondent PHQ-9, all-respondent GAD-7, student sub-group, and employee sub-group results paragraphs, so that readers can see these variables were tested even when associations were not statistically significant.

Line 229: Reference to “(pre/post)” to be removed and clarified to avoid reader confusion. At one point, 6 months of recall vs. 2 weeks immediately prior to the survey is mentioned earlier in the manuscript. For consistency, repeat those time frames throughout the study to remind the reader to the time frames participants were to recall.

Thank you for the comment! We have updated the materials and methods section to be explicit with how we label the different types of recall that were measured during the survey. We carry this labeling forward across the statistical analysis and results section for consistency. Revised text is displayed below.

Upon providing electronic informed consent, participants completed a multi-faceted, self-administered questionnaire that included several validated instruments, including the Patient Health Questionnaire-9 (PHQ-9)[34] and the Generalized Anxiety Disorder-7 (GAD-7)[35]. Notably, the PHQ-9 and GAD-7 were the only measures requiring retrospective recall. For these instruments, participants reported their mood during two time periods: (1) the two weeks immediately preceding the mass casualty event (pre-MCE recall, approximately 4–5 months prior to survey administration), and (2) the two weeks immediately preceding completion of the questionnaire (recent recall).

Lines 227-234: This is a long section that begins with identifying independent variable, covariates, and ends with “were used as control variables.” Is there a period missing somewhere? Please clarify that which were control variables and that which were not.

Thank you for the comment! We have revised this section for clarity of what was the primary variables of interest and covariates. Revised section below.

A linear mixed effects model with random intercepts for participant was used to examine individual differences in PHQ-9 with primary independent variables of recall (pre-MCE recall/current recall), pre-MCE recall GAD-7 scores, current treatment status for anxiety or depression (yes/no), difference in actual (eCRF) to perceived (Hunt Norm) fitness, log-transformed mean PEDQ, and role (student, student and employee, and employee only). The model also included control variables of participant age, Sex (Male, Female), Sex/Gender Minority (non-SGM versus SGM), race, Latino/Hispanic ethnicity, and location at the time of the event (on-campus, near campus, off-campus).

Line 235 is the clearest explanation of how the survey was presented “between recall (pre-MCE verses current)” – consider using this explainer throughout.

Thank you for the comment and emphasizing that use for consistency. We have now updated the manuscript to maintain that explanation of what the survey measured throughout.

Line 263: Again, the clearest explanation of a measurement within the survey – consider using throughout: “questionnaire within the survey”

Thank you! We have now updated the manuscript to make clear this explanation.

Line 273: Table 1 Participant Demographics. You have included measurement responses along with participant demographics. And even then, you do not include ALL of the measurement response. Perhaps separate the information between simple demographics and questionnaire response means.

Thank you for the comment! We have clarified the table into separate sections for demographics versus responses and also included additional measurement responses which were previously not included.

Line 276: BRAVA! “…pre MCE and recent recall” is the best descriptor I’ve read yet! See if this language can be incorporated throughout for increased clarity.

Thank you! We have now incorporated this language throughout.

Line 285: Yes! “Recall difference” makes your research so much clearer. Thank you!

Thank you! This helps us confirm we are on the right track.

Line 301-302: Increased clarity needed. Perhaps just restating the previous sentence about the relationship between pre-MCE recall GAD-7 and both recall time points for PHQ-9. (Or perhaps I will better understand the results section when I get to the discussion section.) ;)

Thank you! We agree this can be made clearer. Below is the revised text.

The interaction between recall period and pre-MCE GAD-7 score was significant (β = -1.09, 95% CI = [-1.83, -0.35], p = .006, Figure 2A): participants with lower pre-MCE GAD-7 scores showed the largest increases in recent-recall PHQ-9, while participants with higher pre-MCE GAD-7 scores showed little change.

Line 309: Typo? “Furthermore there was also a treatment…” Instead of treatment, interaction?

That is correct! We have updated for clarity.

Furthermore, there was also an anxiety and depression by recall interaction

Line 315: Replace “pre/post”

Thank you for catching this! We have updated it to our current terminology for consistency and clarity. Pre-MCE recall/recent recall.

Line 392-393: This feels important to restate “…employees who reported higher perceived discrimination also had higher [anxiety]…”

Thank you! We have revised to make clear what the measure represents for better interpretation.

Lines 440-446: This feels like a significant finding. Consider adding the information from lines 392-393.

Thank you for the comment! We have reiterated the finding within the discussion to better elaborate the results of each analysis. Revises text can be seen below.

However, the magnitude of the difference was dependent on the level of anxiety and depression experienced before the shooting, perceived discrimination, and participation in treatment for anxiety and/or depression. Specifically, among students perceived sense of scho

Attachment Submitted filename: PLOS notes 5 1 26 Final.docx

# Peer review: Decision Letter 2

**DOI:** 10.1371/journal.pone.0353453.r005

**Authors:** Janet Rosenbaum, Janet Rosenbaum, Jiaci Lin

25 Jun 2026

Supporting recovery: Intersections of health, belonging, and help seeking after a campus mass casualty event

PONE-D-25-33879R2

Dear Dr. Jalene,

We’re pleased to inform you that your manuscript has been judged scientifically suitable for publication and will be formally accepted for publication once it meets all outstanding technical requirements.

Within one week, you’ll receive an e-mail detailing the required amendments. When these have been addressed, you’ll receive a formal acceptance letter and your manuscript will be scheduled for publication.

An invoice will be generated when your article is formally accepted. Please note, if your institution has a publishing partnership with PLOS and your article meets the relevant criteria, all or part of your publication costs will be covered. Please make sure your user information is up-to-date by logging into Editorial Manager at Editorial Manager® and clicking the ‘Update My Information' link at the top of the page. For questions related to billing, please contact billing support.

If your institution or institutions have a press office, please notify them about your upcoming paper to help maximize its impact. If they’ll be preparing press materials, please inform our press team as soon as possible -- no later than 48 hours after receiving the formal acceptance. Your manuscript will remain under strict press embargo until 2 pm Eastern Time on the date of publication. For more information, please contact onepress@plos.org.

Kind regards,

Jiaci Lin

Academic Editor

PLOS One

Additional Editor Comments (optional):

I have carefully evaluated the manuscript and considered the comments provided by the reviewers. Both reviewers found the manuscript suitable for publication.

I am therefore pleased to recommend the manuscript for acceptance.

Thank you for choosing PLOS ONE for the publication of your work. Congratulations to you and your co-authors.

Reviewers' comments:

Reviewer's Responses to Questions

**Comments to the Author**

1. If the authors have adequately addressed your comments raised in a previous round of review and you feel that this manuscript is now acceptable for publication, you may indicate that here to bypass the “Comments to the Author” section, enter your conflict of interest statement in the “Confidential to Editor” section, and submit your "Accept" recommendation.

Reviewer #2: All comments have been addressed

Reviewer #3: All comments have been addressed

**********

2. Is the manuscript technically sound, and do the data support the conclusions?

The manuscript must describe a technically sound piece of scientific research with data that supports the conclusions. Experiments must have been conducted rigorously, with appropriate controls, replication, and sample sizes. The conclusions must be drawn appropriately based on the data presented.

Reviewer #2: Yes

Reviewer #3: Partly

**********

3. Has the statistical analysis been performed appropriately and rigorously?

Reviewer #2: Yes

Reviewer #3: Yes

**********

4. Have the authors made all data underlying the findings in their manuscript fully available?

The PLOS Data policy requires authors to make all data underlying the findings described in their manuscript fully available without restriction, with rare exception (please refer to the Data Availability Statement in the manuscript PDF file). The data should be provided as part of the manuscript or its supporting information, or deposited to a public repository. For example, in addition to summary statistics, the data points behind means, medians and variance measures should be available. If there are restrictions on publicly sharing data—e.g. participant privacy or use of data from a third party—those must be specified.

Reviewer #2: Yes

Reviewer #3: Yes

**********

5. Is the manuscript presented in an intelligible fashion and written in standard English?

PLOS ONE does not copyedit accepted manuscripts, so the language in submitted articles must be clear, correct, and unambiguous. Any typographical or grammatical errors should be corrected at revision, so please note any specific errors here.

Reviewer #2: Yes

Reviewer #3: Yes

**********

6. Review Comments to the Author

Please use the space provided to explain your answers to the questions above. You may also include additional comments for the author, including concerns about dual publication, research ethics, or publication ethics. (Please upload your review as an attachment if it exceeds 20,000 characters)

Reviewer #2: Thank you for the opportunity to review the additional revisions. Your attention to detail and responsiveness to feedback makes this a more readable and understandable work.

Reviewer #3: (No Response)

**********

7. PLOS authors have the option to publish the peer review history of their article (what does this mean?). If published, this will include your full peer review and any attached files.

If you choose “no”, your identity will remain anonymous but your review may still be made public.

**Do you want your identity to be public for this peer review?**  For information about this choice, including consent withdrawal, please see our Privacy Policy.

Reviewer #2: No

Reviewer #3: **Yes:**  Caterina G. Roman

**********

# Editor report: Acceptance letter

**DOI:** 10.1371/journal.pone.0353453.r006

**Authors:** Janet Rosenbaum, Janet Rosenbaum, Jiaci Lin

PONE-D-25-33879R2

PLOS One

Dear Dr. Jalene,

I'm pleased to inform you that your manuscript has been deemed suitable for publication in PLOS One. Congratulations! Your manuscript is now being handed over to our production team.

At this stage, our production department will prepare your paper for publication. This includes ensuring the following:

* All references, tables, and figures are properly cited

* All relevant supporting information is included in the manuscript submission,

* There are no issues that prevent the paper from being properly typeset

You will receive further instructions from the production team, including instructions on how to review your proof when it is ready. Please keep in mind that we are working through a large volume of accepted articles, so please give us a few days to review your paper and let you know the next and final steps.

Lastly, if your institution or institutions have a press office, please let them know about your upcoming paper now to help maximize its impact. If they'll be preparing press materials, please inform our press team within the next 48 hours. Your manuscript will remain under strict press embargo until 2 pm Eastern Time on the date of publication. For more information, please contact onepress@plos.org.

You will receive an invoice from PLOS for your publication fee after your manuscript has reached the completed accept phase. If you receive an email requesting payment before acceptance or for any other service, this may be a phishing scheme. Learn how to identify phishing emails and protect your accounts at https://explore.plos.org/phishing.

If we can help with anything else, please email us at customercare@plos.org.

Thank you for submitting your work to PLOS ONE and supporting open access.

Kind regards,

PLOS ONE Editorial Office Staff

on behalf of

Dr. Jiaci Lin

Academic Editor

PLOS One
